# Serotonin-releasing agents with reduced off-target effects

**DOI:** 10.1038/s41380-022-01843-w

**Published:** 2022-11-09

**Authors:** Felix P. Mayer, Marco Niello, Daniela Cintulova, Spyridon Sideromenos, Julian Maier, Yang Li, Simon Bulling, Oliver Kudlacek, Klaus Schicker, Hideki Iwamoto, Fei Deng, Jinxia Wan, Marion Holy, Rania Katamish, Walter Sandtner, Yulong Li, Daniela D. Pollak, Randy D. Blakely, Marko D. Mihovilovic, Michael H. Baumann, Harald H. Sitte

**Affiliations:** 1 Center for Physiology and Pharmacology, Institute of Pharmacology, Medical University of Vienna, Waehringer Strasse 13a, 1090 Vienna, Austria; 2 Department of Biomedical Science, Charles E. Schmidt College of Medicine, Florida Atlantic University, Jupiter, FL 33458 USA; 3 Institute of Applied Synthetic Chemistry, TU Wien, Vienna, Austria; 4 Department of Neurophysiology and Neuropharmacology, Medical University of Vienna, Vienna, Austria; 5 Stiles-Nicholson Brain Institute and Department of Biomedical Science, Charles E. Schmidt College of Medicine, Florida Atlantic University, Jupiter, FL 33458 USA; 6 IDG McGovern Institute for Brain Research, Peking University, 100871 Beijing, China; 7 Designer Drug Research Unit, Intramural Research Program, National Institute on Drug Abuse, National Institutes of Health, Baltimore, MD 21224 USA; 8 AddRess, Center for Addiction Research and Science, Medical University of Vienna, Vienna, Austria; 9 Present Address: Institutes of Brain Science, Fudan University, Shanghai, 200032 China

**Keywords:** Neuroscience, Drug discovery

## Abstract

Increasing extracellular levels of serotonin (5-HT) in the brain ameliorates symptoms of depression and anxiety-related disorders, e.g., social phobias and post-traumatic stress disorder. Recent evidence from preclinical and clinical studies established the therapeutic potential of drugs inducing the release of 5-HT via the 5-HT-transporter. Nevertheless, current 5-HT releasing compounds under clinical investigation carry the risk for abuse and deleterious side effects. Here, we demonstrate that *S*-enantiomers of certain ring-substituted cathinones show preference for the release of 5-HT ex vivo and in vivo, and exert 5-HT-associated effects in preclinical behavioral models. Importantly, the lead cathinone compounds (1) do not induce substantial dopamine release and (2) display reduced off-target activity at vesicular monoamine transporters and 5-HT_2B_-receptors, indicative of low abuse-liability and low potential for adverse events. Taken together, our findings identify these agents as lead compounds that may prove useful for the treatment of disorders where elevation of 5-HT has proven beneficial.

## Introduction

Inhibitors of the serotonin (5-HT) transporter (SERT), also known as 5-HT selective reuptake inhibitors (SSRIs), are commonly used for the treatment of several pervasive conditions including depression and anxiety-related disorders that include social phobia and post-traumatic stress disorder, as well as premature ejaculation [1, 2]. SSRIs act by inhibiting the reuptake of 5-HT, thereby increasing the extracellular concentrations of the neurotransmitter in the brain [3]. Under normal circumstances, SERT mediates high-affinity reuptake and efficient clearance of extracellular 5-HT and thus is a principal regulator of 5-HT transmission in the central nervous system (CNS) and periphery. Unfortunately, the response rate to 5-HT reuptake inhibitors is highly variable, occasioned by dose-limiting side effects for many, and typically associated with a delayed onset of clinical improvement [4]. The delayed onset of efficacy has been attributed to autoinhibitory feedback control—established through 5-HT_1A_ autoreceptors (5-HT_1A_AR), which are required to desensitize following chronic treatment with 5-HT reuptake inhibitors for therapeutic efficacy [5, 6].

Ongoing efforts to improve mood disorder therapeutics aimed at overcoming the limitations of SSRIs include the development of reuptake inhibitors that target other monoamine systems, such as dopamine (DA) and norepinephrine (NE) or that do so in combination with SERT antagonism, as well as mixed action molecules that combine SERT inhibition with actions at 5-HT receptors [3, 7]. Of note, drugs that bind to SERT and the closely related monoamine transporters for DA (DAT) and NE (NET) can be subdivided into two major classes: (1) non-transportable inhibitors (e.g., 5-HT reuptake inhibitors such as SSRIs or cocaine), which prevent reuptake of extracellular monoamines and (2) transportable substrates or “releasers” (e.g., (±)3,4-methylenedioxymethamphetamine (MDMA)) that are actively transported by SERT, DAT), and NET) and subsequently trigger the release of intracellular monoamines by reversing the normal direction of transporter flux [8]. Importantly, the transporter-mediated release of monoamine transmitters occurs independent of ongoing neuronal activity, suggesting that transporter releasers can achieve rapid elevation in extracellular 5-HT and circumvent inhibitory feedback mechanisms that suppress 5-HT neuron excitation [8].

Recent clinical data support the notion that SERT releasers have therapeutic utility. For example, the ring-substituted amphetamine MDMA displayed efficacy for the treatment of PTSD in a recent double-blind, placebo-controlled, phase 3 clinical study [9]. As demonstrated in preclinical models, the prosocial therapeutic effect of MDMA relies on SERT-mediated release of 5-HT in the nucleus accumbens (NAc), a brain structure that is involved in the regulation of reward and social behavior [10]. Importantly, MDMA also induces the release of DA by DAT, which is not required for prosocial effects [10], but contributes to the abuse potential of such agents. Indeed, substantial evidence shows that activity of a given drug at SERT serves to counteract the abuse-related effects that are linked to activity at DAT [11–13], i.e., increasing the relative potency of a given drug at SERT versus DAT reduces its abuse potential. Other examples of 5-HT releasers are fenfluramine (FEN) and its active isomer d-fenfluramine (D-FEN). FEN and D-FEN were approved for clinical use as anorectic drugs but were withdrawn from the market due to adverse effects [14]; FEN has been recently re-approved for the treatment of Dravet syndrome [15].

Taken together, converging lines of evidence suggest that drugs which preferentially increase 5-HT in a transporter-mediated manner may be able to target an unmet medical need.

In our efforts to identify SERT-preferring releasing agents, we chose β-keto amphetamine-derived drugs (i.e., cathinone-derived drugs) as a starting scaffold based on the following rationale: (1) the existing literature has identified basic structure-activity-relationships (SAR) for cathinone compounds [16]; (2) stereochemistry dictates the pharmacological profile of monoamine release induced by cathinones at SERT, but not DAT [17–20], and (3) preclinical data indicate a reduced neurotoxic potential for cathinone-derived drugs when compared to their amphetamine counterparts e.g., MDMA [21, 22]. Apart from widely prescribed medications, e.g., bupropion [23], cathinone-derived compounds are also recreationally consumed new psychoactive substances [16]. However, the abuse liability of the cathinone compounds derives from their DAT activity [24] which can be significantly reduced by increasing their relative activity at SERT [12].

**Fig. 1:**
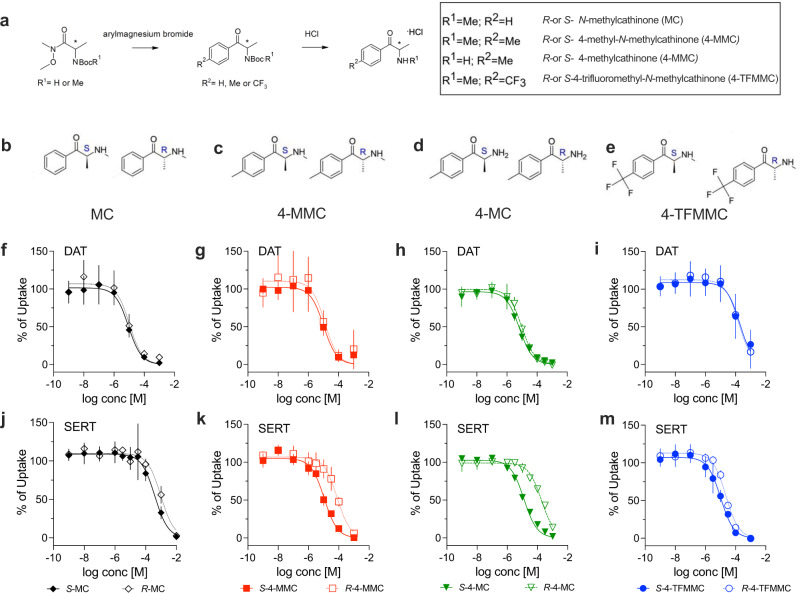
Chemical structures and inhibition of transporter-mediated uptake by cathinones. **a**–**e** display the synthesis and the chemical structures of the stereoisomers of MC, 4-MMC, 4-MC and 4-TFMMC, respectively. **f**–**i** display inhibition of human DAT-mediated uptake of [^3^H]MPP^+^ and **j**–**m** depict inhibition of [^3^H]5-HT transport via human SERT in HEK293 cells. Data are shown as mean and standard deviation. *N* > 3 independent experiments. Detailed sample sizes are given in the Supplementary Information.

Here we report the identification of drugs that preferentially increase 5-HT in vivo in a SERT-dependent manner. Based on an SAR-driven approach, we tested both stereoisomers of *N-*methylcathinone (MC), 4-methyl-*N*-methylcathinone (4-MMC), 4-methylcathinone (4-MC) and 4-trifluoromethyl-*N-*methylcathinone (4-TFMMC) (Fig. 1a–e) for their ability to interact with SERT and DAT. In vitro assays confirmed that all tested cathinones act as substrate-type releasers at DAT and SERT. As shown previously [12, 25], addition of substituents to the *para-*position (i.e., 4-position) on the phenyl ring of MC enhanced the relative selectivity for SERT versus DAT. In accordance with previous observations, we found that the *S*-enantiomers of each drug were several-fold more potent than the corresponding *R*-enantiomers as releasers at SERT. In subsequent behavioral assays, we identified two main compounds, *S*-4-MC and *S*-4-TFMMC, that exerted 5-HT-dependent effects at doses that did not support stimulant-type (i.e., DA driven) properties. Finally, microdialysis and fiber-photometry in freely moving mice confirmed that *S*-4-MC and *S*-4-TFMMC increase extracellular 5-HT levels in vivo via SERT-mediated reverse transport without effect on extracellular DA.

## Materials and methods

### Materials

The synthesis of test drug stereoisomers is described in detail in the Supplementary Information. Tritiated 5-HT ([^3^H]5-HT, 28.3 µCi per mM) and tritiated 1-methyl-4-phenylpyridinium ([^3^H]MPP^+^, 80–85 µCi per mM) were obtained from Perkin Elmer (Boston, MA, USA) and American Radiolabeled Chemicals (St. Louis, MO, USA), respectively. Fluoxetine hydrochloride was from Tokyo Chemical Industry Co., Ltd. (TCI America, Portland, OR, USA). All other reagents and chemicals were purchased from Sigma-Aldrich (St. Louis, MO, USA) unless otherwise noted. To monitor 5-HT release in vivo with fiber photometric recordings, we utilized genetically encoded G-protein-coupled receptor (GPCR)-activation-based 5-HT (GRAB_5-HT_) [26] (AAV9-hSyn-5-HT2h, WZ Biosciences Inc., Columbia, MD, USA) and DA sensors [27] (GRAB_DA_; AAV-hSyn-DA2m (serotype 9) (Vigene Biosciences, Inc., Rockville, MD USA)). HTR2B-Tango was a gift from Bryan Roth (Addgene plasmid # 66410; http://n2t.net/addgene:66410; RRID:Addgene_66410). pGP-CMV-GCaMP6s was a gift from Douglas Kim & the GENIE Project (Addgene plasmid # 40753; http://n2t.net/addgene:40753; RRID:Addgene_40753). 5-HT2A and 5-HT2C receptor constructs were a gift from Prof. Herrick-Davis (Center for Neuropharmacology and Neuroscience, MC-136 P4000, Albany Medical College, New York, 12208-3479). pcDNA4-TO, pcDNA6-TR, pcDNA3.1 were obtained from Invitrogen (Thermo Fisher, Waltham, MA, USA).

### Animals

All in vivo experiments were performed in adult mice (>6 weeks old). For the photometry and microdialysis experiments, which were carried out in Florida, male C57BL/6J mice from The Jackson Laboratory (Bar Harbor, ME, USA) were group-housed in polycarbonate cages (GM500, Tecniplast, Buguggiate, Italy) with lights on from 7 a.m. to 7 p.m. Food and water were provided *ad libitum*. Photometry and microdialysis experiments were performed in accordance with a protocol approved by the Institutional Animal Care and Use Committee at Florida Atlantic University. For the behavioral experiments, which were carried out in Vienna, C57BL/6N mice from Charles River (Sulzfeld, Germany) were group-housed in polycarbonate cages with lights on from 7 a.m. to 7 p.m. Food and water were provided *ad libitum*. Behavioral experiments were performed in accordance with a protocol approved by the Austrian national ethical committee on animal care and use (Bundesministerium für Wissenschaft und Forschung: BMWFW-66.009/0016-WF/V/3b/2015). All animal procedures were conducted in agreement with the ARRIVE guidelines and the U.K. Animal (Scientific Procedures) Act, 1986, and associated guidelines, EU Directive 2010/63/EU for animal experiments. Mice were given at least 1 week to acclimate before being used in any experiments.

### Behavior

For behavioral assays, animals were assigned to treatment groups and injected with test drugs by an unblinded observer.

### Forced-swim test (FST)

The FST was performed as previously described [28]. In brief, mice were placed for 6 min in a beaker (diameter: 19 cm, height: 23 cm), halfway filled with water (22–23 °C), and the immobility time was quantified using the software Videotrack (Viewpoint, Champagne au Mont d’Or, France). Immobility was defined as the absence of any movement, except for those required to keep the head above the water. The time spent immobile during the final 4 min of the test was used as a measure of behavioral despair. Animal sample size was confirmed using a post hoc analysis of the achieved power using G*Power3.1 [29]. Using a significance level (*α*) of 0.05 and an effect size *f* = 2.5 and a sample size of 6 animals/group we achieved the commonly accepted statistical power (1−*β*) of 0.8.

### Open-field test

Mice were placed into an open field arena (27.3 × 27.3 × 27.3 cm) surrounded by infrared beams to track movements. The total distance traveled over 1 h was recorded using Activity Monitor (Med Associates Inc., St. Albans, VT, USA) and interpreted as a measure of the psychostimulant effects. Animal sample size was confirmed using a post hoc analysis of the achieved power using G*Power3.1 [29]. Using a significance level (*α*) of 0.05 and an effect size *f* = 1.92 and a sample size of 7.5 animals (5–10)/group we achieved the commonly accepted statistical power (1−*β*) of 0.8.

### Cell culture

Human embryonic kidney 293 (HEK293) cells stably expressing the human isoforms of either DAT or SERT were maintained in Dulbecco’s Modified Eagle Medium (DMEM), supplemented with 10% fetal calf serum at a subconfluent state in humidified atmosphere (5% CO_2_, 37 °C).

### DAT and SERT uptake inhibition assays in HEK293 cells

Uptake inhibition assays were performed as described earlier [30]. Briefly, HEK293 cells stably expressing human SERT or DAT were seeded onto poly- D-lysine (PDL) coated 96-well plates (40,000 cells well^−1^) the day before an experiment. Prior to the uptake inhibition assay, DMEM was replaced with Krebs-HEPES buffer (KHB, 25 mM HEPES, 120 mM NaCl, 5 mM KCl, 1.2 mM CaCl_2_, 1.2 mM MgSO_4_, and 5 mM D-glucose, pH 7.3) and the cells were preincubated with test drugs for 5 min at room temperature. Uptake assays were initiated by adding 20 nM [^3^H]MPP^+^ to DAT cells or 100 nM [^3^H]5-HT to SERT cells. After 180 s (DAT) or 60 s (SERT), the tritiated substrate was removed by aspiration. Cells were washed once with ice-cold KHB, then lysed in 1% sodium dodecyl sulfate (SDS) and subjected to liquid scintillation counting to quantify tritium accumulated in each sample. Non-specific uptake was determined in the presence of mazindol (1 µM) for DAT or paroxetine (1 µM) for SERT and subtracted throughout.

### [^3^H]5-HT release assays in HEK293 cells

HEK293 cells stably expressing SERT were seeded onto PDL-coated glass coverslips (5 mm diameter) that were placed into 96-well plates at a density of 40,000 cells per well the day before the experiment. Cells were preloaded with [^3^H]5-HT (0.4 µM, 20 min at 37 °C). Subsequently, the cells were transferred into small chambers with a total volume of 200 µl and superfused with KHB at a flow rate of 0.7 ml per min for 40 min to establish a stable baseline before the collection of 2-min fractions was initiated. For each experiment, three basal fractions were collected before the cells were exposed to various concentrations (given in the figure legends) of the test drugs for five fractions. Subsequently, the cells were superfused with 1% SDS for three final fractions that served to determine the total amount of radioactivity present within the cells at the end of each experimental run. The amount of tritium within each fraction was determined by liquid scintillation counting, and release was expressed as percentage of radioactivity released in relation to the total radioactivity present at the beginning of that fraction [30]. Observations from superfusion experiments were excluded in case of premature exposure to SDS.

### VMAT uptake inhibition assays in PC12 cells

VMAT uptake inhibition assays were conducted as previously described, with some modifications [31]. In brief, 10^7^ PC12 cells per well were seeded in 24-well plates the day before experimentation. The cells were pretreated with 100 nM nisoxetine and mazindol to inhibit non-specific binding. PC12 cells were then preincubated with 200 µM digitonin to permeabilize membranes and were kept at room temperature on a plate shaker at low velocity for 30 min. Cells were treated with test drugs and shaken for another 10 min. Tritiated substrate (0.05 µM [^3^H]5-HT) was added, and the cells incubated on the plate shaker for 30 min. To terminate uptake, cells were transferred into 1.5 ml Eppendorf tubes, centrifuged at 5000 rpm for 5 min. The supernatant was aspirated, cells were washed with cold KHB, recentrifuged and lysed with 200 µl SDS. The solutions were transferred into vials containing 2 ml scintillation cocktail and radioactivity was determined via beta-scintillation counting (Perkin Elmer, Waltham, MA, USA). For analysis, inhibitory potency of test drugs was normalized to uptake inhibition caused by the potent VMAT inhibitor reserpine.

### 5-HT receptor-dependent induction of GCamP6 fluorescence in HEK293 cells

#### Cloning of expression plasmids

All cloning steps were performed using NEB Builder (New England Biolabs, Frankfurt/Main, Germany). GCamP6s was cloned into pcDNA3.1. GCaMP is a genetically encoded calcium indicator which allows measurement of calcium flux. To allow for visual inspection of co-expressed 5-HT2 receptors, the enhanced cyan fluorescent protein (eCFP) was added to the C-Terminus of the 5-HT2 receptors. The resulting constructs were then cloned into pcDNA4-TO to allow tetracycline dependent expression.

#### Establishment of stable cell lines

In a first step, HEK293 cells were transfected with pcDNA6-TR and cells stably expressing the Tet-repressor were selected by the addition of blasticidin (60 µg ml^−1^). In a second step, one of the resulting clones was transfected with one of the 5-HT2 receptor constructs and stable clones were selected by the addition of zeocin (150 µg ml^−1^). Suitable clones were selected by visual inspection of the eCFP signal at the plasma membrane. In a last step, the eCFP positive clones were transfected with GCamp6s, and stable clones were selected using G418 (250 µg ml^−1^). The resulting clones were sorted via FACS to isolate GCaMP6s (green fluorescent protein) positive cells and the resulting polyclonal cell lines were used for further experiments. Cells were maintained in DMEM supplemented with 2% fetal bovine serum, blasticidin, zeocin, and G418 at concentrations described above. To induce receptor expression, tetracycline (1 µg ml^−1^) was added to the medium 18–24 h prior to the experiment.

#### In vitro Ca^2+^ Imaging

Imaging experiments were performed at room temperature (22–27 °C). Medium was exchanged for KHB 10 min prior to the experiment and cells were continuously superfused with KHB, using a fast superfusion device (DAD12, ALA Scientific, Farmingdale, NY, USA). Test drugs (0.1, 1, 10 µM) or 5-HT (1 µM) were applied for 30 s, interleaved by KHB superfusion for 300 s. GCamp6s fluorescence was monitored using an inverted microscope (Nikon Eclipse Ti2) equipped with a Nikon ×40 WI (NA 1.25) objective (Nikon Europe, Amsterdam, Netherlands). Fluorescence was excited using a 470 nm LED (Coolled pe4000, Coolled, Andover, NY, USA). Excitation light was filtered through a 480/20 nm bandpass filter and reflected via 505 nm dichroic mirror. Emission light was filtered using a 535/25 nm optical bandpass filter (Nikon Europe, Amsterdam, Netherlands). Images were taken at a frequency of 1 image s^−1^ with an exposure time of 20–30 ms using an sCMOS camera (Andor Zyla 5.5, Oxford Instruments, Abingdon, UK) and NIS-Elements 5.2 software (Laboratory Imaging, Praha, Czech Republic).

#### Image analysis

Image Stacks were background corrected by subtracting the mean intensity of one region of interest (ROI) per image from that of a region devoid of cells. For each image the mean fluorescence intensity of 1–5 ROIs consisting of several clustered cells was measured. The change in intensity of the GCamP6s fluorescence was calculated as the intensity at the end of test drug application minus the intensity before substance application, normalized to the increase of fluorescence evoked by the application of 1 µM 5-HT. Data represent 1–5 fields of view from three independent experiments

#### Confocal microscopy and image analysis

Confocal microscopy and image analysis was conducted as previously described [32]. In brief, cells expressing yellow fluorescent protein(YFP)-tagged human SERT or DAT, respectively, were seeded on PDL-coated 35 mm glass-bottom dishes 24 h before image acquisition. Culture medium was removed and the cells were exposed to the substances of interest (10 µM: *S*-4-MC, *R*-4-MC, *S*-4-MMC, *S*-4-TFMMC, *R-*4-TFMMC. 30 µM: *R*-4-MMC) for 1 h. Subsequently, trypan blue (0.4 %) was added for 10 min. Cells were washed with KHB and kept in buffer during image acquisition. A Nikon A1R+ laser scanning confocal microscope system with a ×60 NA 1.4 oil immersion objective (Nikon, Vienna, Austria) was used for imaging. Trypan blue was excited at 561 nm and the YFP-tagged transporter with a 488 nm laser line. Emitted light was filtered appropriately and detected with a GaAsP PMT detector. Four to five images were taken on 3 separate days. Fiji ImageJ 1.53c was used for image analysis. For each image, two regions of interest were hand-drawn per cell. One encompassed the cell membrane (defined by trypan blue staining) and one the cell interior. Membrane transporter expression levels were calculated as the ratio of membrane versus intracellular mean intensity.

#### Measurement of SERT-mediated currents

Whole-cell patch clamp in heterologous systems is a tool for measuring the transport of drugs by sodium-dependent transporters and therefore identifying their substrates [33, 34]. Transporter-mediated currents were measured by means of whole-cell patch clamp in HEK293 cells stably expressing SERT. Cells were voltage-clamped (−60 mV) and continuously superfused with a physiological external solution that contained 140 mM NaCl, 2.5 mM CaCl_2_, 2 mM MgCl_2_, 20 mM glucose and 10 mM HEPES, pH = 7.4. The pipette solution contained 133 mM potassium gluconate, 6 mM NaCl, 1 mM CaCl_2_, 0.7 mM MgCl_2_, 10 mM HEPES, 10 mM EGTA, pH = 7.2. Currents were measured at room temperature (20–24 °C) using an Axopatch 700B amplifier and pClamp 11.2 software (MDS Analytical Technologies, Sunnyvale, CA, USA). Solutions were applied using a DAD-12 superfusion system and an 8-tube perfusion manifold (ALA Scientific Instruments, Farmingdale, NY, USA), which allowed for rapid solution exchange. Current traces were filtered at 1 kHz and digitized at 10 kHz using a Digidata 1550 (MDS Analytical Technologies). Current amplitudes in response to application of test compounds or 5-HT were quantified using Clampfit 10.2 software (Molecular Devices, San Jose, CA, USA). Transporter-mediated currents elicited by test drugs were normalized to the current amplitude elicited by a saturating concentration of 5-HT (10 µM) applied to the same cell, in order to account for differences in cell expression. For analysis, passive holding currents were subtracted, and the traces were filtered using a 100-Hz digital Gaussian low-pass filter.

#### Stereotaxic surgery

Mice were anesthetized with isoflurane (5% induction, 2% maintenance) and placed into a stereotaxic frame (Kopf Instruments, Tujunga, CA, USA) oriented in the flat skull position. Ophthalmic ointment was applied to prevent drying of the eyes. Ketoprofen (10 mg kg^−1^) was administered subcutaneously (s.c.), whereas bupivacaine and lidocaine were administered locally (s.c.; 100 µl of sterile saline containing 0.05% bupivacaine and 0.2% lidocaine) to the surgical site atop the head. A midline incision was made through the skin on the dorsal surface of the head to expose the skull. Fascia was removed, coordinates for the NAc were determined relative to bregma: anterior/posterior = 1.54 mm; medial/lateral = 0.7 mm; dorsal/ventral = −4.1 mm. A 1 mm burr hole was drilled through the skull. For the mice used in microdialysis, a 5 mm guide cannula (S-5000, Synaptech Inc., Marquette, MI, USA) was slowly lowered through the burr hole into the NAc and secured with glass ionomer cement. For the mice used for fiber photometry, 4*10^12^ genome copies of AAV9-hSyn-5-HT2h or 10^10^ genome copies of AAV-hSyn-DA2m were delivered into the NAc at an infusion rate of 100 nl min^−1^ (total volume of 1 µl) using a 34-gauge needle attached to a 10 µl Nanofil microsyringe (Hamilton, Reno, NV, USA), as previously described [35]. Following injection of the virus, an optical fiber was lowered through the burr hole and secured with glass ionomer cement. Three 1.6 mm screws (00-96X1/16 39052, Plastics One Inc., Roanoke, VA, USA) were affixed to the skull to support a cement stage that was created to secure the implanted guide cannulae or optical fiber. Mice were singly housed following surgery and allowed 6 days of recovery for the microdialysis experiments or 21 days for the photometry experiments. Food and water were available *ad libitum*.

#### In vivo microdialysis

Microdialysis was performed based on a previous publication [36]. After recovery from surgery, mice were placed into a clear cylindrical MTANK W/F enclosure (Instech, Plymouth Meeting, PA, USA) with bedding and water/food available *ad libitum*. A microdialysis probe with an active membrane length of 1 mm (S-5010, Synaptech Inc., Marquette, MI, USA) was inserted into the guide cannula and perfused with artificial cerebrospinal fluid (aCSF; 149 mM NaCl, 2.8 mM KCl, 1.2 mM CaCl_2_, 1.2 mM MgCl_2_, and 5.4 mM D-glucose, pH 7.2) at a flow rate of 1 µl per min. Dialysate samples were collected on ice and stored at −80 °C until they were analyzed using high-performance liquid chromatography with electrochemical detection. Drug-induced changes in extracellular DA and 5-HT were expressed as fold change compared to basal monoamine levels obtained during the first three pretreatment samples.

#### Fiber photometry

To monitor light emitted from the G-protein coupled receptor-activation-based 5-HT (GRAB_5-HT_) and DA (GRAB_DA_) sensors, fiber photometry was performed as described [35]. Briefly, a light emitting diode (465 nm) (CLED_465, Doric Lenses, Quebec, QC, Canada), reflected through a dichroic mirror, was coupled to an optical fiber (200 µm core/225 cladding diameter; Thorlabs, Newton, NJ, USA) that was glued to a metal ferrule (Doric Lenses, Quebec, QC, Canada) and implanted into the NAc as described above. Emitted, band-pass filtered light (500–550 nm, FMC6, Doric Lenses, Quebec,QC, Canada) was detected with a photodetector (Newport Femtowatt silicone PIN; New Focus, San Jose, CA, USA). Data were recorded using a RZ5P lock-in amplifier (Tucker-Davis Technologies, Alachua, FL, USA) controlled with Synapse software. Sinusoidal excitation was delivered at 210 Hz by an LED driver Doric Lenses, LEDD_4) at low power mode. A demodulated signal was low-pass filtered at 6 Hz and digitized at 1017 Hz. Data were analyzed and processed using OriginPro (OriginLab, Northampton, MA, USA), Prism 9 (GraphPad, San Diego, CA, USA) and Microsoft Excel (Microsoft, Redmond, WA, USA). To quantify the effect of test drugs on GRAB_5-HT_ and GRAB_DA_ dependent fluorescence, the signal was normalized to the average of the fluorescence as indicated in the corresponding figures, saline controls were fitted with a one phase decay and the traces were corrected accordingly. Mice were placed into a clear cylindrical MTANK W/F chamber (Instech, Plymouth Meeting, PA, USA) and received test drugs via intraperitoneal injection at the timepoints indicated in the figures and figure legends.

#### Data analysis

All data were analyzed using GraphPad Prism 9 (GraphPad Inc., L Jolla, CA, USA). The statistical tests used are given in each figure legend. Statistical significance was set at *p* ≤ 0.05. Data are shown as the mean and standard deviation. Homoscedasticity was assessed using Brown–Forsythe test. Detailed sample sizes are given in the Supplementary Information.

## Results

### *S*-isomers are more potent inhibitors of SERT versus DAT

First, we tested the effects of the stereoisomers of MC, 4-MC, 4-MMC, and 4-TFMMC on DAT- and SERT-mediated uptake. As shown in Fig.1, each compound acted as a fully efficacious inhibitor of uptake in HEK293 cells stably expressing SERT or DAT. At DAT, the potency of the stereoisomers of each drug was comparable, with IC_50_ values in the low micromolar range for MC, 4-MC and 4-MMC (Fig. 1f–h and Table 1). In line with SARs, the addition of the trifluoromethyl group to the 4-position on the phenyl ring improved the potency at SERT relative to DAT (Table 1). This effect was mainly due to a pronounced rightward-shift at DAT for 4-TFMMC [12, 25, 37], with IC_50_ values exceeding 100 µM at DAT (Fig. 1i and Table 1). In the case of *S*-4-MMC and *S*-4-MC, the addition of a methyl-group to the 4-position increased the potency at SERT and rendered the substances nonselective with respect to DAT and SERT (DAT/SERT ratio = 1.06 and 1.9, respectively; Table 1). In agreement with previous studies on cathinones [17–20], we detected a leftward-shift for all the *S*-enantiomers when compared to the corresponding *R*-enantiomers (Fig. 1j–m and Table 1) at SERT, which further improved the relative potency at SERT versus DAT (all DAT/SERT ratios are summarized in Table 1).

**Table 1:** Effect of test drugs on DAT and SERT-mediated uptake.

IC_50_ (µM) (95% CI in brackets)			
	DAT	SERT	DAT/SERT ratio
*S*-MC	9.1 (6.98–11.67)	392.8 (306.3–503.6)	43.2
*R*-MC	10.62 (6.88–16.6)	963.7 (700.6–1332)	90.7
*S*-4-MMC	10.3 (6.74–16.00)	10.87 (9.18–12.93)	1.06
*R*-4-MMC	12.5 (6.84–23.78)	77.06 (59.63–100.2)	6.2
*S*-4-MC	6.65 (5.83–7.61)	12.63 (11.22–14.23)	1.9
*R*-4-MC	9.6 (8.1–11.41)	185.7 (166.3–207.6)	19.3
*S*-4-TFMMC	190.5 (120.7–310.7)	10.22 (7.9–13.34)	0.05
*R*-4-TFMMC	153.1 (112.7–210.9)	22.64 (19.24–26.7)	0.15

Given are the IC_50_ values (mean and 95 CI) obtained from non-linear regression fits as shown in Fig. 1. DAT/SERT ratios were determined using the following equation: (1/DAT_IC50_)/(1/SERT_IC50_). Higher values indicate higher DAT-selectivity.

### *S*-isomers preferentially release [^3^H]5-HT via SERT

**Fig. 2:**
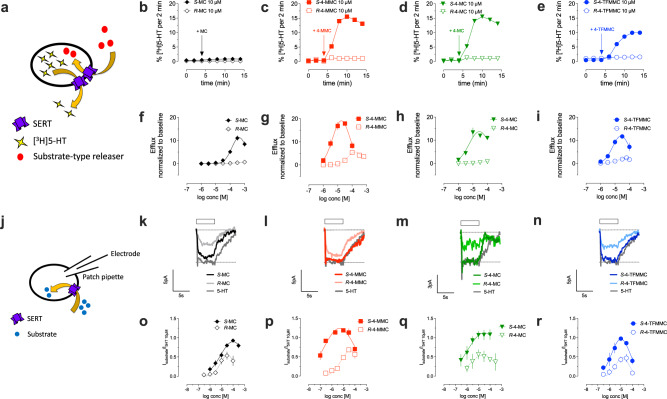
Cathinone-induced 5-HT efflux and electrophysiology. **a** Schematic representation of substrate-type releaser induced efflux of preloaded [^3^H]5-HT from HEK293 cells stably expressing human SERT. Representative traces showing the effect of the two stereoisomers of **b** MC, **c** 4-MMC, **d** 4-MC and **e** 4-TFMMC on SERT-mediated efflux at 10 µM. **f**-**i** Cathinone-induced efflux at *t* = 8–14 min was normalized to basal efflux at *t* = 0–4 min and plotted against the applied concentrations of the stereoisomers of **f** MC, **g** 4-MMC, **h** 4-MC, **i** 4-TFMMC. **j** Schematics of whole-cell patch clamp experiments used to identify cathinone-induced inwardly directed currents in SERT-expressing HEK293 cells. Representative single-cell traces showing currents elicited by 10 µM of **k** *S-* and *R*-MC, **l** *S-* and *R*-4-MMC, **m** *S-* and *R*-4-MC, **n** *S-* and *R*-4-TFMMC. **o**–**r** Cathinone-induced currents were normalized to the current elicited by bath-application of 10 µM 5-HT and plotted against the applied concentrations. Data in **f**–**i** and **o**–**r** are shown as mean and standard deviation. Data shown in **d** and **h** are replotted from 10.1016/j.neuropharm.2018.12.032. *N* > 3 independent observations per compound and concentration. Detailed sample sizes are given in the Supplementary Information.

Data from uptake inhibition assays, as depicted in Fig. 1, identify the concentrations at which drugs interact with the transporter, but these assays cannot identify the specific mechanism of drug interaction, i.e., non-transportable inhibitor versus transportable substrate (i.e., releaser). To ascertain the potential of the test drugs to promote transporter-mediated release of cytosolic substrates, SERT-expressing HEK293 cells were preloaded with [^3^H]5-HT and exposed to increasing concentrations of the *S*- and *R*- enantiomers of MC, 4-MC, 4-MMC and 4-TFMMC (Fig. 2a). Representative traces of cathinone-triggered 5-HT release are shown in Fig. 2b–e: addition of 10 µM *R*-MC, *R-*4-MC, *R-*4-MMC and *R-*4-TFMMC did not alter basal release of 5-HT (Fig. 2b–e). In contrast, application of 10 µM of *S-*4-MC, *S-*4-MMC and *S*-4-TFMMC robustly increased the amount of [^3^H]5-HT in the superfusate (Fig. 2c-e). *S*-MC had little effect on [^3^H]5-HT release at 10 µM (Fig. 2b), in agreement with its known selectivity for DAT. We tested a series of increasing concentrations for each individual enantiomer to determine their potency as 5-HT releasers (Supplementary Fig. 1a–f). Each *S*-enantiomer evoked [^3^H]5-HT-release in a concentration-dependent manner (Fig. 2f–i). Remarkably, in terms of [^3^H]5-HT-release, the *R*-enantiomers displayed both a rightward-shift and reduced efficacy when compared to the corresponding *S*-enantiomers (Fig. 2f–i).

Transporter-mediated release of preloaded [^3^H]monoamines occurs by reversal of normal transporter flux and serves as an indirect readout of actively transported substrate drugs via monoamine transporters [30]. To measure SERT-mediated transport of the drugs of interest across cellular membranes and to evaluate the impact of stereochemistry of MC, 4-MC, 4-MMC and 4-TFMMC thereon, we performed electrophysiological recordings. Monoamine transporters utilize the sodium gradient across cell membranes as a driving force to concentrate their substrates in the cytosolic compartment [38]. Consequently, SERT-mediated transport of substrate-type drugs gives rise to an inwardly directed current carried by sodium cations, rendering the recording thereof a decisive tool for identifying substrates. We performed whole-cell patch clamp recordings (Fig. 2j) with HEK293 cells stably expressing SERT to determine drug-induced SERT-mediated currents. As shown in Fig. 2k–r, the currents observed upon bath-application of the *S*-enantiomers of MC, 4-MC, 4-MMC and 4-TFMMC exceeded those elicited by the corresponding *R*-enantiomers in terms of amplitude. Consistent with the stereoselective effects on [^3^H]5-HT-release observed for the *S*- and *R-*enantiomers, the electrophysiological recordings revealed a reduction in transporter-mediated current for each *R*-enantiomer. Linear regression analysis revealed a strong positive correlation (*R*^2^ = 0.9661; *F* = 170.9; *p* < 0.0001) between the maximal induced current and release of [^3^H]5-HT (Supplementary Fig. 1g), indicating these two phenomena are closely related. Addition of selected substituents to the 4-position on the phenyl ring of synthetic cathinones afforded the generation of drugs with improved selectivity at SERT versus DAT. Furthermore, stereoselectivity uniquely impacted substrate activity at SERT.

### *S*-isomers display weak effects at VMATs and 5-HT receptors

It is widely accepted that monoaminergic neurotoxicity produced by psychostimulants involves interaction of drug molecules with the vesicular monoamine transporter 2 (VMAT2), thereby evoking the release of intracellular monoamines from synaptic vesicles into the cytosol [39]. Here, we performed uptake inhibition assays in PC12 cells which endogenously express VMATs. We found that *S*-4-MC and *S*-4-TFMMC were significantly less effective as inhibitors of VMAT-mediated transport when compared to MDMA, whereas no difference between *S*-4-MMC and MDMA was detected in this regard (Supplementary Fig. 2a). Deleterious side effects of the 5-HT releasing agents FEN and D-FEN led to their withdrawal from the clinical market. More specifically, administration of FEN or D-FEN induces valvular heart disease that is linked to activation of cardiac 5-HT_2B_ receptors by their *N*-dealkylated metabolites [40]. Accordingly, we tested *S*-4-MC and *S*-4-TFMMC for their activity at 5-HT2 receptor subtypes. At 1 µM, neither test drug activated 5-HT_2A_, 5-HT_2B_, or 5-HT_2C_ receptors. At 10 µM, *S*-4-MC displayed activity at the 5-HT_2A_ receptor and moderate effects at the 5-HT_2B_ receptor (Supplementary Fig. 2b, c). Various studies show that compounds that interact with SERT and DAT can affect the surface expression of both transporters [41–45]. We found that treatment with the stereoisomers of 4-MC, 4-MMC and 4-TFMMC did not affect the surface expression of SERT and DAT, respectively (Supplementary Fig. 3).

### *S*-4-MC and *S*-4-TFMMC reduce behavioral despair

**Fig. 3:**
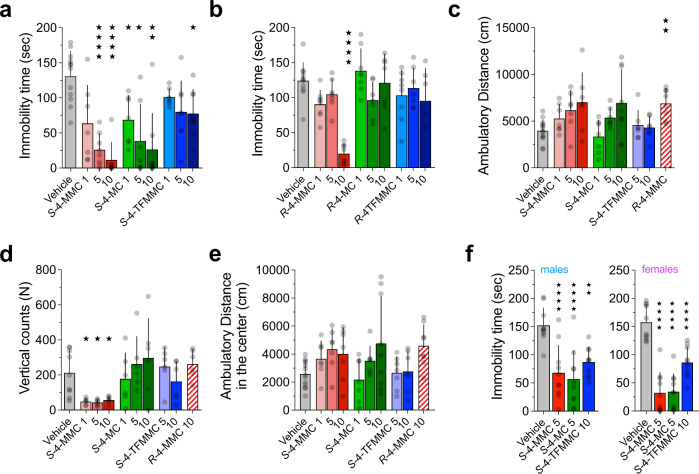
Stereospecific effect of cathinones on behavior. Total time spent immobile in the forced-swim test following systemic (intraperitoneal) administration of vehicle or **a** *S*-4-MMC, *S*-4-MC and *S*-4-TFMMC or **b** *R*-4-MMC, *R*-4-MC and *R*-4-TFMMC at the indicated doses (1, 5 and 10 mg kg^−1^). **c** Total distance traveled, **d** vertical counts and **e** distance traveled in the center in the open field test following intraperitoneal injections of *S*-4-MMC, *S*-4-MC, *S*-4-TFMMC at the indicated doses (1, 5 and 10 mg kg^−1^) and *R*-4-MMC (10 mg kg^−1^). **f** Total time spent immobile in the forced-swim test in male and female mice after systemic administration (intraperitoneal) of *S*-4-MMC (5 mg kg^−1^), *S*-4-MC (5 mg kg^−1^) and *S*-4-TFMMC (10 mg kg^−1^). Data are shown as the mean and standard deviation (bars) with the individual mice being represented by gray symbols. **a**, **c**–**e** were analyzed using Brown–Forsythe and Welch ANOVA, followed by Dunnet’s T3 multiple comparison test. **b**, **f** were analyzed with ordinary one-way ANOVA and Dunnett’s multiple comparison test to identify differences versus vehicle. *p ≤ 0.05; **p ≤ 0.01; ***p ≤ 0.001; ****p ≤ 0.0001. *N* = at least 6 animals per compound and dose. Detailed sample sizes are given in the Supplementary Information.

Our in vitro experiments showed that *S*-enantiomers of ring-substituted cathinones display improved DAT/SERT ratios in uptake inhibition and release assays, and they provided a series of compounds having identical physical-chemical properties but different substrate/blocker profiles at SERT (*S*- versus *R*-enantiomers). To understand if the improved DAT/SERT ratio and the different substrate/blocker profile were sufficient to impact serotonin-related behaviors we tested *S*- and *R*-enantiomers of MC, 4-MC, 4-MMC and 4-TFMMC in the forced-swim test (FST), an established behavioral test for evaluating behavioral despair in rodents [46]. Based on the moderate shift in the DAT/SERT ratio of *S-*MC versus *R*-MC (Fig. 1 and Table 1), which points to little or no improvement in the abuse liability of MC, we decided to investigate only 4-MC, 4-MMC and 4-TFMMC in our behavioral studies. We tested each of the stereoisomers for their potential to reduce the time spent immobile in the FST, a widely accepted paradigm to identify serotonergic effects of test drugs [47]. Relative to vehicle, acute injection of the *S*-enantiomer of 4-MC, 4-MMC, or 4-TFMMC dose-dependently decreased immobility time (Fig. 3a). *S-*4-MMC significantly reduced immobility time versus vehicle at 5 and 10 mg kg^−1^ and *S-*4-MC significantly reduced immobility time versus vehicle at all concentrations tested, i.e., 1, 5 and 10 mg kg^−1^. In case of *S-*4-TFMMC only the highest dose (10 mg kg^−1^) significantly differed from the vehicle group (Fig. 3a). The *R*-enantiomers of 4-MC and 4-TFMMC (1, 5 and 10 mg kg^−1^) had no effect in the FST, whereas the highest dose of *R-*4-MMC (10 mg kg^−1^) significantly reduced immobility time (Fig. 3b).

In the open field test, we observed that *R*-4-MMC, which has been shown to induce robust elevations in ambulatory activity [17] significantly affected horizontal locomotor activity at 10 mg kg^−1^. In contrast, *S*-4-MC and *S*-4-MMC and *S*-4-TFMMC did not differ from vehicle (Fig. 3c).

Only *S*-4-MMC affected vertical counts (Fig. 3d) and no significant effects were observed for ambulatory activity in the center of the test arena (Fig. 3e). To identify potential sex-specific effects of the test drugs, we included both male and female mice in the FST. *S*-4-MC, *S*-4-MMC (both at 5 mg kg^−1^) and *S*-4-TFMMC (10 mg kg^−1^) significantly reduced the time spent immobile regardless of sex (Fig. 3f). Based on the higher SERT selectivity of 4-MC and 4-TFMMC when compared to 4-MMC [25, 48], we chose to examine the stereoisomers of 4-MC and 4-TFMMC in more detail in vivo. Moreover, because *S*-4-MMC was similar to MDMA in its efficacy to inhibit VMAT-mediated transport at 30 µM, we chose to omit this drug from further characterization.

### *S*-4-MC and *S*-4-TFMMC release 5-HT, but not DA, in vivo

**Fig. 4:**
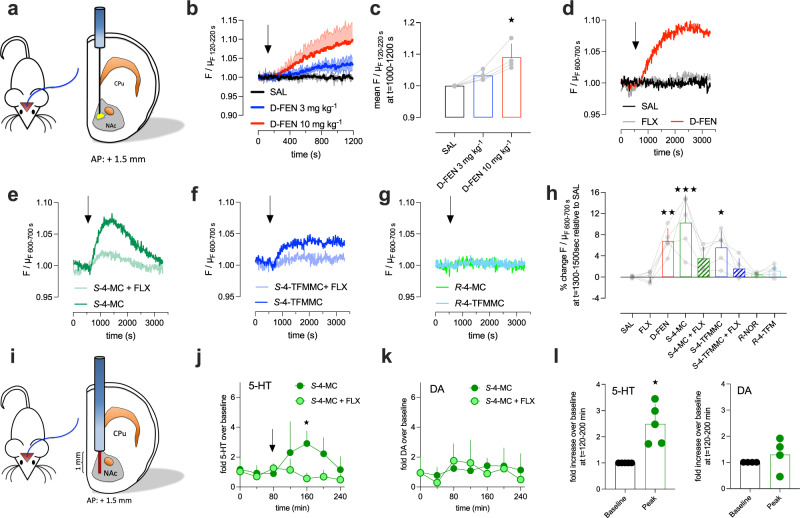
Stereospecific effect of cathinones on SERT-dependent 5-HT release in vivo. **a** Schematics of optic probe placement in the NAc of freely moving mice to assess extracellular 5-HT using a genetically encoded sensor. CPu = caudate putamen. **b** Representative single traces displaying the effect of saline (SAL) or D-FEN (3 or 10 mg kg^−1^, intraperitoneal injections at t = 120 s) on the relative fluorescence emitted by the 5-HT sensor. Data are shown as mean and standard deviation (*N* = 4 per condition). **c** Comparison of the effect of 3 and 10 mg kg^−1^ D-FEN on the sensor-emitted fluorescence versus saline control. Representative single traces showing changes in sensor-derived fluorescence following intraperitoneal injections (at t = 600 s) of **d** SAL, fluoxetine (FLX, 10 mg kg^−1^), D-FEN (10 mg kg^−1^), **e** *S*-4-MC (5 mg kg^−1^) plus vehicle (=*S*-4-MC, dark green) or *S*-4-MC (5 mg kg^−1^) plus FLX (10 mg kg^−1^) (=*S*-4-MC + FLX, light green), **f** *S*-4-TFMMC (10 mg kg^−1^) plus vehicle (=*S*-4-TFMMC, dark blue) or *S*-4-TFMMC (10 mg kg^−1^) plus FLX (10 mg kg^−1^) (=*S*-4-TFMMC + FLX, light blue) and **g** *R*-4-MC (5 mg kg^−1^) and *R*-4-TFMMC (10 mg kg^−1^). **h** Comparison of drug induced changes in fluorescence (*N* = 5 per condition). **i** Cartoon depicting guide cannula (blue column) and active microdialysis membrane (red tip, active length of 1 mm) placement in the NAc. **j**, **k** Effect of intraperitoneal *S*-4-MC (5 mg kg^−1^) on extracellular 5-HT and dopamine (DA) in presence or absence of co-administered fluoxetine (FLX, 10 mg kg^−1^) or vehicle. * denotes *p* ≤ 0.05 (Bonferroni’s) at the corresponding timepoint **l** fold increase in extracellular 5-HT and DA at *t* = 160 min relative to baseline (*t* = 0–80 min) as shown in **j** and **k**. One-sample *t*-test versus the hypothetical mean of 1 (*p ≤ 0.05). Data are shown as mean and standard deviation. Data in **b**, **c**, **h**, **j**, **k** and **l** are given as mean and standard deviation. Data in **c** and **h** were analyzed using Kruskal–Wallis, followed by Dunn’s multiple comparison test. *p ≤ 0.05, **p ≤ 0.01 and ***p ≤ 0.001 versus SAL. Detailed sample sizes are given in the Supplementary Information.

In preclinical studies, the prosocial effect of MDMA strictly requires SERT-mediated release of 5-HT in the NAc [10]. Blockage of SERT with SSRIs blunts the prosocial effect of MDMA in animal models [10] and antagonizes subjective effects (including positive mood and extraversion) of MDMA in humans [49, 50]. Consequently, we tested the effect of *S*-4-MC and *S*-4-TFMMC on extracellular 5-HT in vivo. Recently, genetically encoded fluorescent probes that allow for the detection of 5-HT were developed [26]. We delivered GPCR-activation-based 5-HT (GRAB_5-HT_) sensors into the NAc and implanted optic probes (Fig. 4a) to perform fiber photometry in freely moving mice. First, to confirm that the sensor-derived fluorescence was sensitive to changes in extracellular 5-HT, mice were treated with the known 5-HT-releasing agent D-FEN. Intraperitoneal injection of D-FEN (3 and 10 mg kg^−1^) increased the basal fluorescence in a dose-dependent manner (Fig. 4b, c and Supplementary Fig. 4), which confirmed the utility of this approach to monitor the effect of 4-MC and 4-TFMMC on extracellular 5-HT. Representative traces are displayed in Fig. 4d–g. As shown in Fig. 4d, D-FEN (10 mg kg^−1^) increased 5-HT when compared to saline (SAL), whereas acute administration of the SSRI fluoxetine (10 mg kg^−1^) was without effect. Both *S*-4-MC (5 mg kg^−1^) and *S*-4-TFMMC (10 mg kg^−1^) elevated extracellular 5-HT in the NAc in a time-dependent manner, and this effect could be prevented by fluoxetine (Fig. 4e, f). In line with in vitro data, no effect on 5-HT was observed after administration of *R*-4-MC (5 mg kg^−1^) or *R*-4-TFMMC (10 mg kg^−1^). Based on previous reports [25, 48], and the time course observed in Fig. 4e, we quantified the relative fluorescence at *t* = 700–900 s post injection to determine the effect of the test drugs at the peak of their effects. Significant effects were observed for D-FEN, *S*-4-MC and *S*-4-TFMMC alone, but not in combination with fluoxetine (Fig. 4h; individual recordings are shown in Supplementary Fig. 5).

Finally, to confirm the validity of the results obtained with fiber photometry, the effect of fluoxetine on cathinone-evoked 5-HT release in the NAc was assessed with microdialysis in freely moving mice (Fig. 4i-l). First, we ensured that two doses of *S*-4-MC (5 mg kg^−1^) elicited comparable changes in 5-HT when given 24 h apart. Mice that expressed the genetically encoded 5-HT sensor in the NAc were injected with *S*-4-MC on 2 consecutive days and no significant difference was detected between the drug effect on day 1 and day 2 (Supplementary Fig. 6). On day 1 of microdialysis, mice were injected with *S*-4-MC (5 mg kg^−1^) after the collection of three basal dialysates. On the next day, mice received fluoxetine (FLX, 10 mg kg^−1^) in combination with *S*-4-MC (5 mg kg^−1^). As shown in Fig. 4j, application of repeated measures two-way ANOVA (drug treatment × time) revealed that drug-treatment significantly affected 5-HT in the dialysate (*F*_1, 8_ = 17.62; *p* = 0.0030), but not DA (*F*_1, 6_ = 0.1497; *p* = 0.7122; Fig. 4k). In addition, *S*-4-MC significantly elevated 5-HT in the dialysate, but not DA versus baseline (Fig. 4l). Additionally, we employed genetically encoded fluorescent probes that allow for the detection of DA [51] and performed fiber photometric recordings thereof and found that neither *S*-4-MC, nor *S*-4-TFMMC significantly elevated the basal DA levels in the NAc (Supplementary Fig. 7). Finally, as observed for *S*-4-MC, fluoxetine markedly blunted the amount of dialysate 5-HT when mice were injected with *S*-4-TFMMC (Supplementary Fig. 8).

## Discussion

A variety of psychiatric medications target SERT proteins in the brain. For example, SSRIs are commonly used to treat depression and anxiety disorders, social phobias, and premature ejaculation. More recently, the SERT releaser MDMA has shown promise for the treatment of PTSD [9], whereas FEN was recently re-approved in a low-dose formulation for the treatment of Dravet syndrome [15]. Here we examined cathinone-derived compounds as candidate medications targeting SERT. Our study reports three main findings: (1) *S* isomers of ring-substituted cathinones display preferential substrate activity at SERT over DAT in vitro, (2) *S* isomers of 4-MC and 4-TFMMC have minimal activity at VMATs and 5-HT_2B_ receptors, two molecular targets associated with adverse side effects and (3) *S* isomers of 4-MC and 4-TFMMC evoke 5-HT release in vivo and rescue behavioral despair as revealed in the FST, a commonly used paradigm that exhibits predictive validity for the identification of antidepressants [46]. Importantly, the SERT-mediated effects of 4-MC and 4-TFMCC are independent of exocytotic release, suggesting these compounds could have immediate effects on extracellular concentrations of 5-HT in the brain and maintain efficacy despite suppression of 5-HT neuron excitation due to the activation of somatic 5-HT_1A_ autoreceptors that leads to reduced vesicular release. Taken together, our results identify novel potential candidate medications targeting the serotonergic system.

Emerging evidence indicates that MDMA is an efficacious adjunct treatment for PTSD [9], and thus it is tempting to speculate that 5-HT releasers that mimic the serotonergic actions of MDMA might have utility in other psychiatric disorders. Converging lines of evidence reveal that subjective effects of MDMA require reverse transport of 5-HT through SERT [10, 49, 50]. Consequently, this mechanism of action clearly differentiates MDMA from SSRIs that require ongoing vesicular release to enhance the synaptic concentration of 5-HT. Using a heterologous expression system, we found that the *S*-enantiomers of 4-MC, 4-MMC and 4-TFMMC evoke the release of 5-HT through SERT similar to the mechanism of MDMA. Remarkably, the corresponding *R-*enantiomers cause either negligible (4-MC, 4-TFMMC) or blunted (4-MMC) efflux of 5-HT at the concentrations tested. Our in vitro release findings agree with numerous publications showing that ring-substituted cathinones cause the release of 5-HT via SERT in vitro and in vivo, and that stereochemistry of these compounds influences their relative potency at SERT versus DAT [17–20]. Using voltage clamp techniques in cells expressing SERT, we show that the *S*-enantiomers which release 5-HT are able to induce marked SERT-mediated inward currents. Again, this effect is blunted for all the tested *R-*enantiomers. Linear regression analysis revealed that the ability of the compounds to elicit 5-HT efflux is strongly correlated with the corresponding steady-state currents. This observation provides support for the interpretation that drug-induced efflux via SERT is linked to translocation of drug molecules by the transporter. Future studies are warranted to address the biophysical underpinnings that render the tested cathinones in their *R*-configuration less transportable through human SERT. Notably, Hutsell et al. [18] found that *R*-4-MC is a fully efficacious 5-HT releaser in rat brain synaptosomes. Hence, species differences will have to also be considered in follow-up investigations.

One of the potential adverse effects of cathinone-based drugs that target monoamine transporters is abuse liability, mediated chiefly by DA uptake inhibition or DA efflux activity at DAT [16]. Various ring-substituted cathinones are readily self-administered [52, 53]. However, increasing activity at SERT will counteract the abuse-related effects that stem from activity at DAT [12, 16, 54–56]. Here, and in line with previous studies [12], we show that cathinone compounds bearing ring substitutions at the 4-position display preferential effects at SERT over DAT, a feature that was even more pronounced for the corresponding *S*-enantiomers [17–20]. Thus, in vivo use should be titratable to doses where only minimal or no DA efflux is triggered despite substantial 5-HT efflux, as shown in our mouse studies. Taken together, these findings posit that abuse liability will be minimal, and future studies should address this issue.

Many transporter substrates are also associated with monoaminergic neurotoxicity. For example, high dose administration of amphetamines is known to deplete brain tissue monoamines by interacting with VMAT2 which disrupts vesicular storage [39]. When compared to MDMA, cathinone-derived compounds are less neurotoxic in mice and rats [57–59]. One possible explanation for the reduced neurotoxic potential of cathinone-derived compounds is their reduced inhibitory potency at VMAT2 [60, 61]. Here we demonstrate that *S*-4-MC and *S*-4-TFMMC are less effective as inhibitors at VMATs when compared to MDMA, whereas the inhibitory effect of *S*-4-MMC is comparable to that observed for MDMA. However, Pifl et al. reported the potency of racemic 4-MMC to inhibit VMAT2-mediated uptake to be 10-fold lower than that of MDMA [62]. Hence, the present data support the notion that *S*-4-MC and *S*-4-TFMMC, but perhaps not *S*-4-MMC, will have reduced neurotoxic potential due to their weak actions at VMATs. Consequently, their actions will likely (1) not overload the presynaptic cytoplasm with oxidizable 5-HT arising from vesicular depletion nor (2) deplete vesicular storage pools needed to carry out normal excitation-coupled vesicular release of the neurotransmitter once 5-HT_1A_ mediated desensitization has been overcome.

Perhaps the most problematic side-effect of SERT substrates is cardiac valve disease induced by mitogenic effects of 5-HT_2B_ receptor stimulation. The appetite suppressants FEN and D-FEN were removed from clinical use due cardiac valve disease. Studies show that the adverse effects of FEN and D-FEN are due to activation of 5-HT_2B_ receptors by their *N*-dealkylated metabolites [40]. We found that *S*-4-MC had little effect on the 5-HT_2B_ receptor at 1 µM, whereas a moderate activation was observed at 10 µM. Previously, we detected no binding of *S*-4-MC to the 5-HT_2B_ receptor at concentrations as high as 7 µM [19]. However, as low doses of *S*-4-MC are required to elicit 5-HT release in the nervous system (i.e., 5 mg kg^−1^), future studies should address the question of whether 5-HT_2B_ receptors are indeed activated upon administration of *S*-4-MC in vivo. Neither stereoisomer of 4-MC, 4-MMC or 4-TFMMC significantly affected surface expression of DAT or SERT, which further suggests that the compounds identified in this study are associated with limited off-target effects.

Our behavioral findings revealed that systemic administration of the *S*-enantiomers of 4-MC, 4-MMC and 4-TFMMC significantly reduces the time spent immobile in the FST. The decreased immobility time in the FST is thought to indicate antidepressant-like activity [46], which is consistent with serotonergic effects of cathinone-derived compounds. Indeed, compounds that inhibit SERT-mediated uptake reduce the immobility time to a considerable extent [46]. In the cases of citalopram and fluoxetine, both for acute and chronic effects, these actions are lost in mice where the high-affinity interaction between SERT and fluoxetine/citalopram has been disrupted [47] which highlights the contribution of intact SERT function to the mechanism of action of these agents. *R*-4-MC and *R-*4-TFMMC had no effect on the time spent immobile at all doses tested, whereas *R-*4-MMC significantly reduced the immobility time when administered at 10 mg kg^−1^. These findings are in agreement with data gathered from in vitro experiments that revealed a strong rightward-shift for the *R*-enantiomers at SERT. Drug-induced locomotor activity is strongly correlated with the extracellular levels of DA in the CNS [63]. We found that *S*-4-MC, *S*-4-MMC and *S*-4-TFMMC significantly reduced the time spent immobile at doses that did not enhance locomotor activity, which indicates that both drugs exert distinct elevations in extracellular 5-HT at doses that do not recruit DA release. Likewise, the significant effect of 10 mg kg^-1^ of *R-*4-MMC in the FST might be a result of enhanced locomotor activity observed in the current study and a previous report [17]. We did not observe sex-specific effects in the FST, which suggests that the acute effects of *S*-4-MC, *S*-4-MMC and *S*-4-TFMMC are similar in male and female mice.

Considering that the beneficial effects of MDMA appear to result from SERT-dependent release of 5-HT rather than mere inhibition of reuptake [10, 49], we sought to verify that *S*-4-MC and *S*-4-TFMMC indeed increase the extracellular 5-HT levels via reverse transport. Based on the moderate effect of *S*-4-MMC on VMAT-mediated transport and rearing, we excluded *S*-4-MMC from further characterization. We chose to employ fiber photometric recordings of genetically encoded fluorescent sensors for 5-HT in the NAc of freely moving mice in real time. To our knowledge, this is the first study to use this technology to compare and evaluate pharmacological effects of multiple test drugs on basal neurotransmitter levels. The high temporal resolution allows for immediate detection of alterations in basal neurotransmitter levels that appear to be in excellent agreement with the pharmacokinetics of a given drug [35]. Using this technique, we found that *S*-4-MC and *S*-4-TFMMC robustly elevate extracellular 5-HT in the NAc and that this effect is blocked with a SERT inhibitor. The lack of effect of the corresponding *R*-enantiomers confirmed our hypothesis, i.e., that the pro-serotonergic effect of the tested cathinones stems from the activity of the *S*-enantiomers at SERT. Considering the novelty of the sensor approach, we supported our fiber photometry studies using in vivo microdialysis. Again, we found that *S*-4-MC and *S*-4-TFMMC robustly elevate extracellular 5-HT in the NAc and that this effect is blocked with a SERT inhibitor. *S*-4-MC and *S*-4-TFMMC did not affect extracellular DA, which has been shown previously for racemic 4-TFMMC [25]. These observations agree with our behavioral data and ultimately confirm their pharmacological activity as preferential 5-HT releasers.

Collectively, our data indicate that *S*-4-MC and *S*-4-TFMMC bear the potential to modulate the 5-HT system in clinical settings without the undesired side effects of other SERT substrates like MDMA and fenfluramines. Importantly, *S*-4-MC and *S*-4-TFMMC evoke the release of 5-HT independently of vesicular release. We observed this effect in the NAc, a brain structure with pronounced SERT expression that is implicated in the regulation of social behaviors. Enhancing impaired 5-HT transmission in the NAc restores social deficits in a preclinical model for autism [64], and Heifets et al. [10] reported that 5-HT release within the NAc is required for the prosocial effects of MDMA. Hence, both *S*-4-MC and *S*-4-TFMMC might be effective agents to aid social deficits and support the formation of therapist-patient bonds. In addition, the time-course of *S*-4-MC-induced 5-HT release could support its application in settings that require a fast onset scenario, e.g., for the treatment of premature ejaculation, which is associated with a deficiency in extracellular 5-HT [65]. One limitation of SERT inhibitors is that they require high occupancy of SERT in vivo to achieve clinical efficacy, which likely contributes to undesirable side effects and driving efforts to combine other pharmacological agents with reduced SERT antagonism [7]. Based on the in vitro profile of *S*-4-MC and *S-*4-TFMMC, and the doses administered in our in vivo experiments, we suspect that both compounds could induce substantial 5-HT release at subsaturating concentrations, though investigations in human subjects are needed to validate this idea. Future studies shall investigate potential therapeutic effects of *S*-4-MC and *S*-4-TFMMC in preclinical models. Detailed information about the safety profile of cathinones and targeted medicinal chemistry will be employed to further develop drugs that preferentially and rapidly increase 5-HT with reduced abuse liability and side effects.

## Supplementary information

Supplementary Information

Supplementary Information Figure Legends

Supplementary Figure 1

Supplementary Figure 2

Supplementary Figure 3

Supplementary Figure 4

Supplementary Figure 5

Supplementary Figure 6

Supplementary Figure 7

Supplementary Figure 8

## Data Availability

Data supporting the findings of this study are available within the article and its Supplementary Information Files and from the corresponding authors upon reasonable request at http://www.zenodo.org (10.1038/s41380-022-01843-w.).
