# Supplementary material for: Serotonin-releasing agents with reduced off-target effects
Source: Mol Psychiatry. 2022 Nov 9;28(2):722–32. doi: 10.1038/s41380-022-01843-w (PMC9645344; doi:10.1038/s41380-022-01843-w)
Supplement: Supplementary file 1 — Supplementary Information [file 41380_2022_1843_MOESM1_ESM.pdf]

Suppl. Information

Serotonin-releasing agents with reduced off-target effects

Felix P. Mayer*,§,1,2, Marco Niello*,1, Daniela Cintulova[3], Spyridon Sideromenos [4], Julian Maier[1], Yang Li[1#], Simon Bulling[1], Oliver Kudlacek[1], Klaus Schicker[2], Hideki Iwamoto[5], Fei Deng[6], Jinxia Wan[6], Marion Holy[2], Rania Katamish[2], Walter Sandtner[1], Yulong Li[6], Daniela D. Pollak[4], Randy D. Blakely[2;5], Marko D. Mihovilovic[3], Michael H. Baumann[7], Harald H. Sitte[§,1,8]

[1] Center for Physiology and Pharmacology, Institute of Pharmacology, Medical University of Vienna, Vienna, Austria.
[2] Department of Biomedical Science, Charles E. Schmidt College of Medicine, Florida Atlantic University, Jupiter, FL 33458, USA
[3] Institute of Applied Synthetic Chemistry, TU Wien, Vienna, Austria
[4] Center for Physiology and Pharmacology, Institute of Physiology, Medical University of Vienna, Vienna, Austria.
[5] Stiles-Nicholson Brain Institute and Department of Biomedical Science, Charles E. Schmidt College of Medicine, Florida Atlantic University, Jupiter, FL 33458, USA
[6] IDG McGovern Institute for Brain Research, Peking University, Beijing 100871, China.
[7] Designer Drug Research Unit, Intramural Research Program, National Institute on Drug Abuse, National Institutes of Health, Baltimore, MD 21224, USA.
[8] AddRess, Center for Addiction Research and Science, Medical University of Vienna, Vienna, Austria.
[#] Present address: Institutes of Brain Science, Fudan University, Shanghai, 200032, China
*These two authors contributed equally to the current publication.
[§]Address correspondence to:
harald.sitte@meduniwien.ac.at
felixpmayer@gmail.com

Keywords: Serotonin, PTSD, MDMA, drug assisted psychotherapy, serotonin transporter, dopamine transporter

Chemical synthesis

Unless noted otherwise, all reagents were purchased from commercial suppliers and used without further purification. DCM, Et_2O, dioxane, MeOH, THF and toluene intended for water-free reactions were pre-distilled and then desiccated on Al_2O_3 columns (PURESOLV, Innovative Technology). For all other solvents, quality grade is given in the reaction procedures.

NMR spectroscopy

NMR spectra were recorded on a Bruker *AC 200* (^{1}H: 200 MHz, ^{13}C: 50 MHz) and Bruker *Avance Ultrashield 400* (^{1}H: 400 MHz, ^{13}C: 101 MHz) and Bruker *Avance IIIHD 600* spectrometer equipped with a Prodigy BBO cryo probe (^{1}H: 600 MHz, ^{13}C: 151MHz). Chemical shifts are given in parts per million (ppm) and were calibrated with internal standards of deuterium labeled solvents $CDCl_3$ (^{1}H 7.26 ppm, ^{13}C 77.16 ppm), MeOD (^{1}H 3.31 ppm, ^{13}C 49.00 ppm), D_2O (^{1}H 4.80 ppm,^{13}C N/A) and DMSO-d_6 (^{1}H 2.50 ppm, ^{13}C 39.52 ppm). NMR assignments of unknown compounds were confirmed by ^{1}H - ^{1}H COSY, ^{1}H - ^{13}C, HSQC and ^{1}H - ^{13}C, HMBC and by comparison to predicted spectra. Proton multiplicities are denoted by the following abbreviations: s (singlet), brs (broad singlet), d (doublet), dd (doublet of a doublet), ddd (doublet of a doublet of a doublet), t (triplet), dt (doublet of a triplet), q (quartet), dq (doublet of a quartet), p (quintet), hep (septet), m (multiplet). Coupling constants (*J*) are presented in Hz (Hertz). Carbon multiplicities (suppressed CH coupling) are denoted by the following abbreviations: s (singlet), d (doublet), t (triplet) and q (quartet). In case of fluoro structures the coupling constant is denoted generally as "xy,$^zJ_{C,F}$ = …Hz" whereby x represents the multiplicity of the CH coupling, y the multiplicity of the CF coupling and z the order of spin-spin coupling.

Chromatographic methods

TLC was performed using silica gel 60 aluminum plates containing fluorescent indicator from Merck and detected either with UV light at 254 nm or by charring in ninhydrin solution (300 mg ninhydrin, 3 mL acetic acid, 100 mL butanol), 2,4-dinitrophenylhydrazine (0.8 g 2,4- dinitrophenylhydrazine, 200 mL 2N HCl, 2 mL EtOH) or potassium permanganate (1 g $KMnO_4$, 6.6 g K_2CO_3, 100 mg NaOH, 100 mL H_2O in 1M NaOH) with heating.

Column chromatography was performed on a Büchi Sepacore Flash System (2 x Büchi Pump Module C-605, Büchi Pump Manager C-615, Büchi UV Photometer C-635, Büchi Fraction Collector C-660) or standard manual glass columns using silica gel from Merck (40-63 µm) using LP or *n*-hexane and DCM, Et_2O or EtOAc mixtures.

Enantiomeric excess was determined *via* normal phase HPLC with a ChiralPak AS-H (250 mm x 4.6 mm ID) on a Thermo Scientific/Dionex Ultimate 3000 HPLC using mixtures of *n*-hexane/*n*-heptane and *i*PrOH/EtOH. Basic analytes were eluted using 0.1 vol% diethylamine.

Melting point

Melting points were determined by a Büchi Melting Point B-545 device.

Specific rotation

Specific rotation $[\alpha]_D^{20}$ was determined using an MCP 500 polarimeter from Anton Paar by the following equation: $[\alpha]_D^{20}$= 100*α/[c]*l; c in [g/100 mL], l in [dm].

GENERAL PROCEDURES

General procedure A: Preparation of Grignard reagents

A three-neck flask equipped with condenser, thermometer, septum and magnetic stirring bar was evacuated and dried using a hot-air gun. After cooling to room temperature it was flushed with argon and charged with magnesium turnings (1 equiv.). Usually 1 - 2 mL of solvent were added, followed by dropwise addition of neat aryl bromide. Change of color and the formation of an exotherm indicated start of the reaction, upon which the rest of the solvent was added (the amount of solvent was calculated to give the final 1M or 0.5M solution of Grignard reagent). The remaining aryl bromide was slowly added dropwise, and the reaction was stirred until magnesium was fully dissolved.

4-Tolylmagnesium bromide

**Concentration, solvent**	1M in THF
**Reaction time**	Dropwise addition of 1-bromo-4-methylbenzene over 15 min (first at rt, then under cooling), 1h heated to 50 °C
**Appearance**	Dark brown solution

4-Trifluoromethylphenylmagnesium bromide

**Concentration, solvent**	0.5M in THF
**Reaction time**	Dropwise addition of 1-bromo-4-(trifluoromethyl) benzene under cooling<br>45 min at rt.
**Appearance**	Dark red solution

General procedure B: Addition of Grignard reagents to alanine-derived Weinreb amide

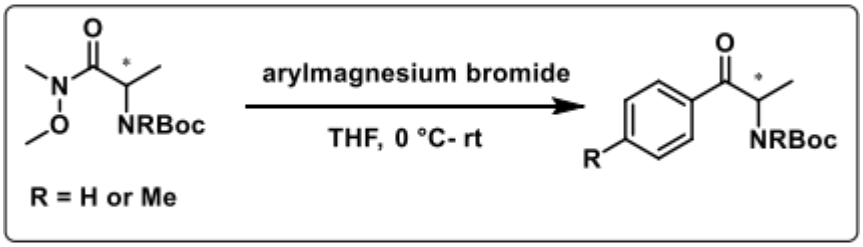

In an oven-dried three-neck round bottom flask equipped with septum, thermometer and magnetic stirring bar, the corresponding Weinreb amide (1 equiv.) was dissolved in dry THF (c = 0.14M) and cooled to 0 °C. A solution of arylmagnesium bromide (1 M or 0.5M, 3 equiv.) was added dropwise. The amount of solvent used to dissolve the starting material was adjusted to give a final concentration of 1 mmol/10 mL. The reaction was maintained at 0 °C for one hour, warmed to rt and stirred for 0.5 - 2 h until full conversion was indicated by TLC. After completion the reaction was cooled to 0 °C and quenched by addition of satd. NH_4Cl and water. The mixture was extracted with three times with Et_2O, dried over anh. $MgSO_4$ and evaporated.

General procedure C: Boc-deprotection

In an 8-mL screw-cap vial Boc-protected compounds (1 equiv.) were dissolved in a small amount of HPLC-grade dioxane and cooled to 0 °C, upon which they were treated with pre-cooled 6 M HCl in H_2O/dioxane = 4/1 (this solution was prepared by mixing 50 mL of conc. HCl and 20 mL of dioxane and diluting the mixture to 100 mL with deionized water) to give a 0.2 M solution. The reaction mixture was stirred at 0 °C for 7 to 24 h. After completion of the reaction a transparent solution was obtained. The reaction mixture was extracted multiple times with Et_2O or EtOAc and the aqueous layer was evaporated in a stream of pressurized air. The residue was taken up in deionized water, filtered through a syringe filter and lyophilized to obtain the desired hydrochlorides.

(*R*/*S*)-*tert*-(Butoxycarbonyl)-alanine

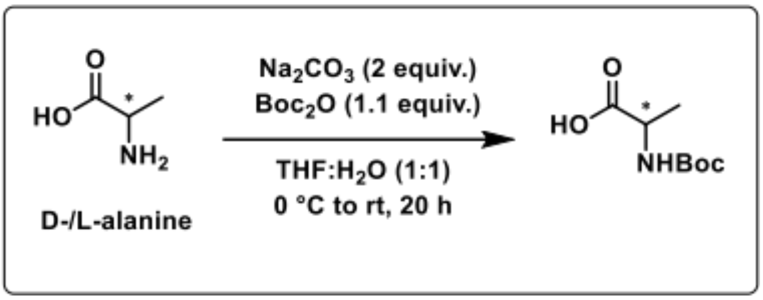

In a round bottom flask (*D*)- or (*L*)-alanine (4 g, 45 mmol, 1 equiv.) was dissolved in 1:1 mixture H_2O:THF (60 mL) along with Na_2CO_3 (9.5 g, 90 mmol, 2 equiv.).The mixture was cooled down to 0 °C and then Boc_2O (10.8 g, 50 mmol, 1.1 equiv.) was slowly added and the reaction was allowed to warm up to rt and stirred for 20 h. Additional 30 mL of water were added to facilitate stirring of otherwise slurry mixture. After this time pH was adjusted to 2 by careful addition of 1M HCl under cooling. The mixture was extracted with EtOAc (4 x 100 mL), combined organic phases were washed with brine, dried over $MgSO_4$ and solvent was removed under reduced pressure, yielding the desired product as colorless solid in **99 %** (*R*), resp. **87 % yield** (*S*). The product was used as such without further purification.

^{1}H NMR (**400 MHz, DMSO-*d*$_6$**) Mixture of rotamers: δ 1.21 (d, *J* = 7.3 Hz, 3H), 1.30 – 1.44 (m, 9H), 3.77 – 3.97(m, 1H), 6.66 – 6.74 (m, 1H), 7.06 (d, *J* = 7.5 Hz, 1H), 12.37 (s, 1H). **^{13}C NMR (101 MHz, DMSO-*d*$_6$)** δ 17.1, 28.2, 48.8, 77.9, 155.3, 174.7; m.p 79 – 82 °C, (*R*): $[\alpha]_D^{20}$ = +25.2° (*c* 1, MeOH), (*S*): α_D^{20}= -24.3° (*c* 1, MeOH).

(*R*/*S*)-*N*-(*tert*-Butoxycarbonyl)-*N*-methyl-alanine

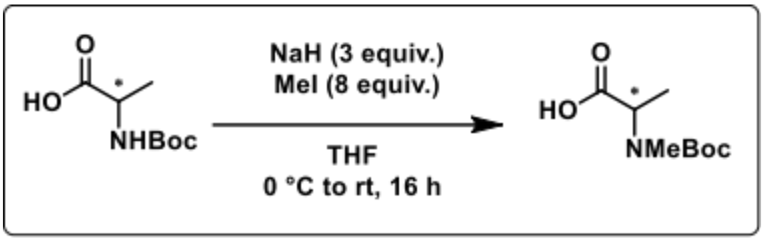

In an oven-dried round bottom flask (*L*)- or (*D*)-*N*-Boc-alanine (2 g, 10.6 mmol, 1 equiv.) was dissolved in dry THF (35 mL) under argon and cooled to 0 °C. Then sodium hydride (1.4 g, 31.8 mmol, 3 equiv., 60% in mineral oil) was added in three portions and the reaction was stirred for 1 h at 0 °C. Then neat MeI (5.3 mL, 84.8 mmol, 8 equiv.) was added dropwise via syringe at 0 °C and the reaction mixture was allowed to warm to room temperature and let stir for 16 h. The reaction was quenched with water (100 mL) and extracted with Et_2O (100 mL). The organic phase was separated and washed with satd. $NaHCO_3$ (80 mL). The aqueous phases were then combined, pH was adjusted to 2 by careful addition of 2M HCl under cooling and extracted with EtOAc (3 x 80 mL). The combined organic layers were then washed with satd. $Na_2S_2O_3$ (3 x 50 mL), dried over anh. $MgSO_4$ and the solvent was evaporated under reduced pressure, furnishing the desired product as colorless solid in **80 %** (*R*), resp. **91 % yield** (*S*). The product was used as such without further purification which was used as such without need for further purification.

^{1}H NMR (400 MHz, DMSO-*d*$_6$) Mixture of rotamers ≈ 60:40: δ 1.24 –1.33 (m, 3H), 1.32 – 1.45 (m, 9H), 2.73 (m, 3H), 4.54 (m, 0.5H), 4.27 (m, 0.5H); **^{13}C NMR (101 MHz, DMSO-*d*$_6$)**: δ 14.6, 15.2, 27.9, 28.0, 30.6, 31.5, 53.2, 54.8, 79.0, 154.6, 155.0, 173.3, 173.4; m.p 89 – 92 °C, (*R*): $[\alpha]_D^{20}$ = +45.6° (*c* 1, DCM), (*S*): α_D^{20}= -42.5° (*c* 92, DCM).

(R)/(S)-*tert*-Butyl (1-(methoxy(methyl)amino)-1-oxopropan-2-yl)(methyl)carbamate

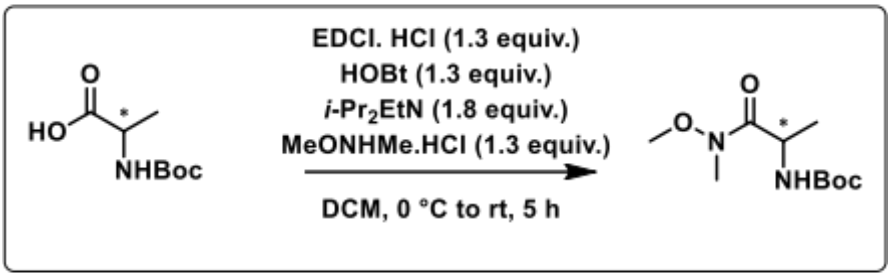

An oven-dried three-neck round bottom flask was charged with (*D*)- or (*L*)-*N*-Boc-alanine (4 g, 21.2 mmol, 1 equiv.) and dry DCM (85 mL) under argon and then 1-hydroxybenzotriazol (4.2 g, 27.5 mmol, 1.3 equiv.), *N,O*-dimethylhydroxylamine hydrochloride (2.7 g, 27.5 mmol, 1.3 equiv.) and *i*-Pr_2EtN (5.5 mL, 31.8 mmol, 1.5 equiv.) were added successively. The reaction mixture was cooled to 0 °C and *N*-ethyl-*N'*-(3-dimethylaminopropyl)carbodiimide hydrochloride (5.3 g, 27.5 mmol, 1.3 equiv.) was added in one portion. The reaction was allowed to warm to rt and stirred for 5 h when TLC indicated full conversion. The reaction was quenched by addition of water (100 mL) and extracted with DCM (3 x 75 mL). The combined organic layers were washed with 1M HCl (100 mL), satd. $NaHCO_3$ (100 mL) and brine (100 mL), dried over anh. $MgSO_4$ and evaporated, yielding the desired product as off-white crystals in **85 %** (*R*), resp. **99 % yield** (*S*). The product was used as such without further purification which was used as such without need for further purification.

^{1}**H NMR (400 MHz, $CDCl_3$)** δ 1.28 (d, *J* = 7.0 Hz, 3H), 1.41 (s, 9H), 3.18 (s, 3H), 3.74 (s, 3H), 4.73 – 4.56 (m, 1H), 5.24 (d, *J* = 8.1 Hz, 1H); ^{13}**C NMR (101 MHz, $CDCl_3$)** 18.8, 28.5, 32.3, 46.63, 61.7, 79.6, 155.3, 173.8; m.p 146 – 147 °C, (*R*): $[\alpha]_D^{20}$ = +28.5° (*c* 0.95, MeOH), (*S*): α_D^{20}= -25.3° (*c* 1, MeOH).

(R)/(S)-*tert*-Butyl (1-(methoxy(methyl)amino)-1-oxopropan-2-yl)(methyl)carbamate

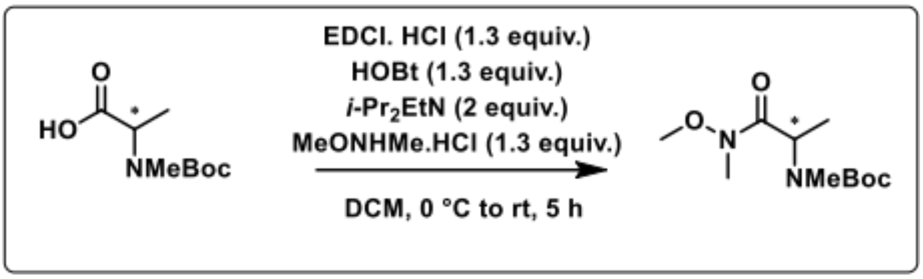

(*D*)- or (*L*)-*N*-Me-*N*-Boc-alanine (1.5 g, 7.4 mmol, 1 equiv.) was dissolved in dry DCM (30 mL) under argon and then 1-hydroxybenzotriazol (1.5 g, 9.6 mmol, 1.3 equiv.), *N,O*-dimethylhydroxylamine hydrochlorid (0.94 g, 9.6 mmol, 1.3 equiv.) and *i*-Pr_2EtN (2.3 mL, 13.3 mmol, 2 equiv.) were added successively. The reaction mixture was cooled to 0 °C and EDCI hydrochloride (1.8 g, 9.6 mmol, 1.3 equiv.) was added. The reaction was allowed to warm to rt and stirred for 5 h. The reaction was quenched by addition of water (50 mL) and extracted with DCM (3 x 50 mL). The combined organic layers were washed with 1M HCl (50 mL), satd. $NaHCO_3$ (50 mL) and brine (50 mL), dried over anh. $MgSO_4$ and evaporated, yielding the desired product as pale yellow oil in **96 %** (*R*), resp. **99 % yield** (*S*). The product was used as such without further purification which was used as such without need for further purification.

^{1}**H NMR (400 MHz, DMSO-*d*$_6$)** Mixture of rotamers ≈ 60:40: δ 1.16 – 1.25 (m, 3H), 1.31 – 1.47 (m, 9H), 2.56 – 2.72 (m, 3H), 3.09 (s, 3H), 3.67 (s, 3H), 4.77 (q, *J* = 6.3 Hz, 0.5H), 4.99 (q, *J* = 6.3 Hz, 0.5H); ^{13}**C NMR (101 MHz, DMSO-*d*$_6$):** δ 14.4, 14.7, 28.0, 29.7, 49.7, 51.2, 61.1, 79.0, 154.4, 154.8,171.9; (*R*): $[\alpha]_D^{20}$ = +57.6° (*c* 1, DCM), (*S*): α_D^{20} = -53.5° (*c* 1.06, DCM).

(*R*)/(*S*)-*tert*-Butyl methyl(1-oxo-1-(p-tolyl)propan-2-yl)carbamate

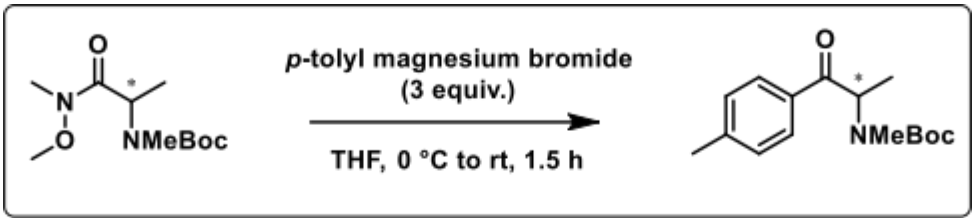

The compound was prepared according to **General procedure B**. The desired compound was obtained after purification (LP/EtOAc = 95/5, 90 g SiO_2) as off-colorless solid in **82%** (*R*), resp. **80%** (*S*) yield.

^{1}H-NMR (400 MHz, DMSO-*d*$_6$): Mixture of rotamers ≈ 50:50: δ 1.18 – 1.43 (m, 12H), 2.36 (s, 3H), 2.62 (s, 1.5H), 2.79 (s, 1.5H), 5.08 (q, *J* = 6.7 Hz, 0.5H), 5.42 (q, *J* = 6.8 Hz, 0.5H), 7.22 – 7.37 (m, 2H), 7.70 – 7.84 (m, 2H);**^{13}C-NMR (101 MHz, DMSO-*d*$_6$)**: Mixture of rotamers ≈ 50:50: δ 13.3, 13.8, 21.1, 27.7, 27.9, 30.2, 31.7, 55.1, 57.3 (d, C2), 79.3, 79.4, 128.0, 129.1, 132.8, 133.1, 143.2, 143.4, 153.8, 154.6, 198.6, 198.7; m.p. 55 – 57 °C, (*R*): $[\alpha]_D^{20}$= 148° (*c* 1.1, DCM), >99% ee, (*S*): $[\alpha]_D^{20}$ = -144° (*c* 0.9, DCM), >99% ee; HPLC method: Hexane/EtOH = 99.7/0.3, 1mL/min, 25 °C, 30min, AS-H.

(*R*)/(*S*)-*tert*-Butyl (1-oxo-1-(p-tolyl)propan-2-yl)carbamate

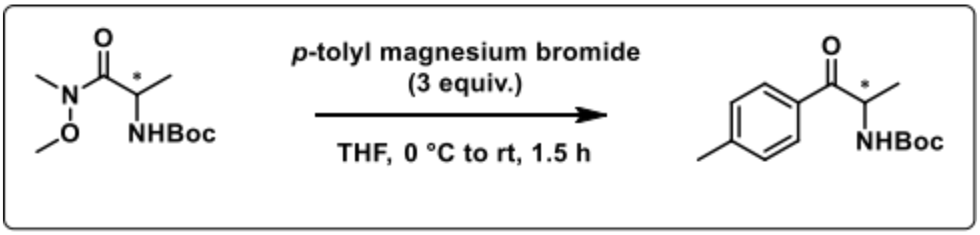

The compound was prepared according to **General procedure B**. The desired compound was obtained after purification (LP/EtOAc = 95/5, 90 g SiO_2, followed by re-crystallization from ligroin) as colorless crystals in **61%** (*R*), resp. **58%** (*S*) yield.

^{1}H-NMR (400 MHz, $CDCl_3$): δ 1.39 (d, *J* = 7.1 Hz, 3H), 1.45 (s, 9H), 2.42 (s, 3H), 5.26 (p, *J* = 7.2 Hz), 5.58 (d, *J* = 7.6 Hz, 1H), 7.28 (d, *J* = 7.7 Hz, 2H), 7.87 (d, *J* = 8.2 Hz, 2H); **^{13}C-NMR (101 MHz, $CDCl_3$)**: δ 20.3, 21.9, 28.5, 51.1, 79.8, 128.9, 129.7 (d, C3' & C5'), 131.8, 144.8, 155.3,199.1; m.p. 101 – 103 °C, (*R*): α_D^{20}= -8.4° (*c* 0.95, DCM), ee n.d., (*S*): $[\alpha]_D^{20}$= +8.7° (*c* 1.2, DCM), ee n.d.

(R)/(S)-*tert*-Butyl methyl(1-oxo-1-phenylpropan-2-yl)carbamate

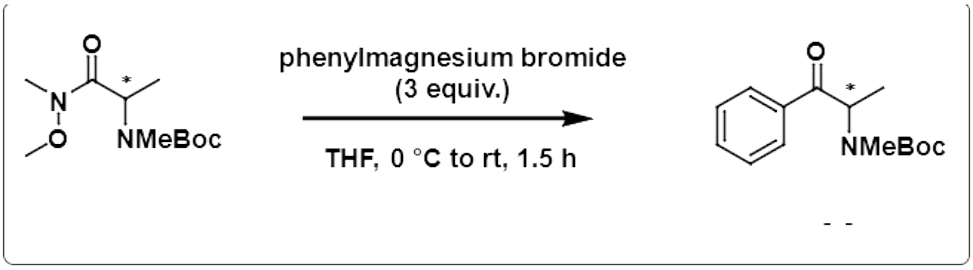

The compound was prepared according to General procedure B (using commercial phenylmagnesium bromide 1M in THF). The desired compound was obtained after purification (LP/EtOAc = 95/5, 90 g SiO2) as colorless oil in **78%** (R), resp. **80%** (S) yield.

^{1}H-NMR (400 MHz, $CDCl_3$): Mixture of rotamers ≈ 60:40 δ 13.4 (q, C3), 13.9 (q, C3), 28.5 (q, tBu), 29.7 (q, N-CH_3), 30.9 (q, N-CH_3), 54.8 (d, C2), 57.2 (d, C2), 80.4 (s, tBu), 80.8 (s, tBu), 128.3 (d, C2' & C6'), 128.6 (d, C2' & C6'), 128.7 (d, C3' & C5'), 133.3 (d, C4'), 135.6 (s, C1'), 155.6 (s, N-CO-O), 200.0 (s, C1);**^{13}C-NMR (101 MHz, $CDCl_3$)**: Mixture of rotamers ≈ 50:50: δ 13.3, 13.8, 21.1, 27.7, 27.9, 30.2, 31.7, 55.1, 57.3 (d, C2), 79.3, 79.4, 128.0, 129.1, 132.8, 133.1, 143.2, 143.4, 153.8, 154.6, 198.6, 198.7; (R): $[\alpha]_{D20}$= +165.1° (c 1, DCM, 99% ee, (S): $[\alpha]_{D20}$ = -162.6° (c 1.05, DCM), 99% ee; HPLC method: Hexane/EtOH = 99.7/0.3, 1mL/min, 25 °C, 30min, AS-H.

(R)/(S)-*tert*-Butyl methyl(1-oxo-1-(4-(trifluoromethyl)phenyl)propan-2-yl)carbamate

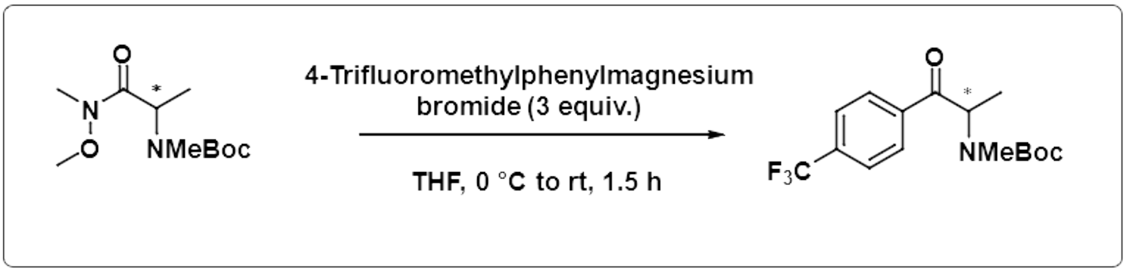

The compound was prepared according to General procedure B (using 4-trifluoromethylphenylmagnesium bromide 0.5M in THF). The desired compound was obtained after purification (LP/EtOAc = 95/5, 90 g SiO_2) as colorless solid in **75%** (R), resp. **81%** (S) yield.

^{1}H-NMR (400 MHz, DMSO-*d_6*): Mixture of rotamers ≈ 50:50 δ 1.08 – 1.47 (m, 12H, *t*Bu & H3), 2.75 (s, 1.5H, N-CH_3), 2.86 (s, 1.5H, N-CH_3), 4.95 – 5.08 (m, 0.5H, H2), 5.18 (q, *J* = 6.0 Hz, 0.5H, H2), 7.81 – 7.95 (m, 2H, H2' & H6'), 7.95 – 8.03 (m, 2H, H3' & H5'); **^{13}C-NMR (101 MHz, DMSO-*d_6*)**: Mixture of rotamers ≈ 50:50 δ 12.7 (q, C3), 13.3 (q, C3), 27.6 (q, *t*Bu), 27.7 (q, *t*Bu), 31.9 (q, N-CH_3), 32.8 (q, N-CH_3), 57.4 (d, C2), 58.7 (d, C2), 79.5 (s, *t*Bu), 79.9 (s, *t*Bu), 123.8 (q, $^1J_{C\text{-}F}$ = 273.5 Hz, CF_3), 125.7 (dq, $^3J_{C\text{-}F}$ = 3.8 Hz, C3' & C5'), 125.9 (dq, $^3J_{C\text{-}F}$ = 3.8 Hz, C3' & C5'), 128.4 (d, C2'), 129.8 (d, C2'), 132.4 (q, $^2J_{C\text{-}F}$ = 32.3 Hz, C4'), 139.4 (s, C1'), 139.7 (s, C1'), 153.6 (s, N-CO-O), 154.4 (s, N-CO-O), 198.7 (s, C1), 198.8 (s, C1) ppm; m.p. 81 – 83 °C, (R): $[\alpha]_{D20}$= +111.8° (*c* 1, DCM), 98% ee, (S): $[\alpha]_{D20}$ = -112.6° (*c* 1, DCM), 98% ee; HPLC method: Hexane/EtOH = 99.7/0.3, 1mL/min, 25 °C, 30min, AS-H.

(*R*)/(*S*)-2-(Methylamino)-1-(p-tolyl)propan-1-one hydrochloride, mephedrone, 4-MMC

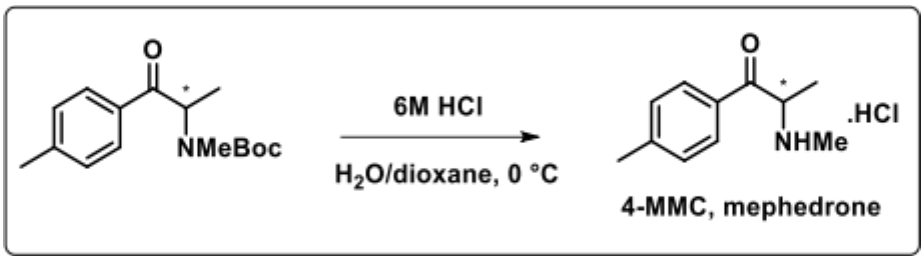

The compound was prepared according to **General procedure C**. The desired compound was obtained as colorless solid in **97%** (*R*), resp. **97%** (*S*) yield.

^{1}H-NMR (400 MHz, D_2O): δ 1.61 (d, *J* = 7.3 Hz, 3H), 2.45 (s, 3H), 2.81 (s, 3H), 5.08 (q, *J* = 7.3 Hz, 1H), 7.46 (d, *J* = 8.1 Hz, 2H), 7.92 (d, *J* = 8.2 Hz, 2H); **^{13}C-NMR (101 MHz, D_2O)**: δ 15.5, 21.0, 31.0, 59.6, 129.0, 129.6, 129.9, 147.4, 197.1 ppm; m.p. decomposition >180 °C, (*R*): α_D^{20}= +40.3° (*c* 0.7, H_2O), 97% ee, (*S*): α_{D20}= -41.5° (*c* 0.8, H_2O), 98% ee; HPLC method: Heptane/IPA = 97/3 + 0.1% DEA, 1mL/min, 25 °C, AS-H (t_S= 8.2 min, t_R= 15.1 min).

(*R*)/(*S*)-2-amino-1-(p-tolyl)propan-1-one, nor-mephedrone, 4-MC

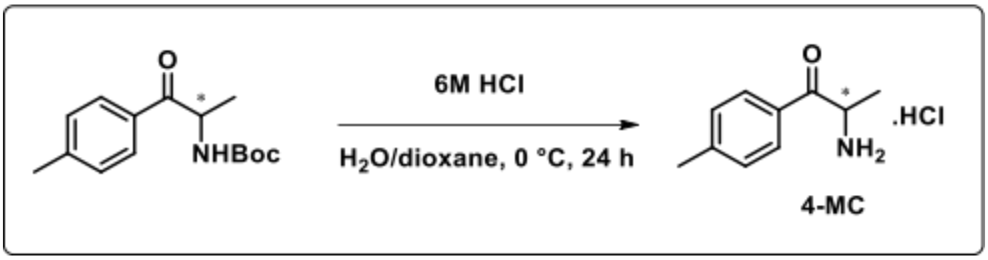

The compound was prepared according to **General procedure C**. The desired compound was obtained as colorless solid in **92%** (*R*), resp. **83%** (*S*) yield.

^{1}H-NMR (400 MHz, D_2O): δ 1.60 (d, *J* = 7.3 Hz, 3H), 2.44 (s, 3H), 5.17 (q, *J* = 7.3 Hz, 1H), 7.44 (d, *J* = 8.0 Hz, 2H), 7.92 (d, *J* = 8.2 Hz, 2H); **^{13}C-NMR (101 MHz, D_2O)**: δ 16.8, 20.9, 51.8, 129.0, 129.6, 129.8, 147.1, 197.6; m.p. decomposition >180 °C, (*R*): α_{D20} = +40.3° (*c* 0.7, H_2O), >99%ee, (*S*): α_{D20} = -42.5° (*c* 0.8, H_2O), >99% ee; HPLC method: Heptane/IPA = 96.9/3 + 0.1% DEA, 1mL/min, 25 °C, AS-H (t_S= 23.9 min, t_R= 28.7 min).

(R)/(S)-2-(Methylamino)-1-phenylpropan-1-one hydrochloride, methcathinone, MCAT

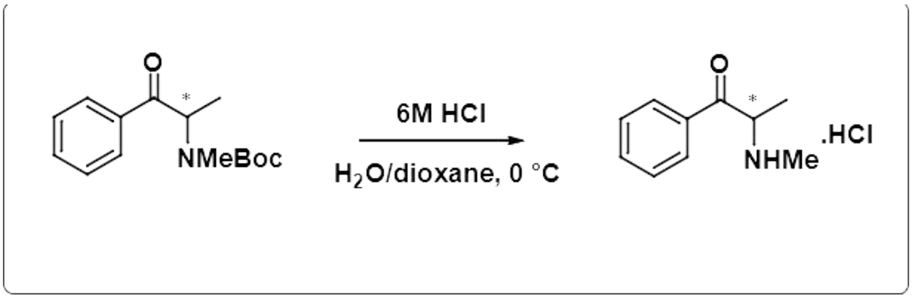

The compound was prepared according to **General procedure C**. The desired compound was obtained as off-colorless solid in **84%** (R), resp. **100%** (S) yield.

^{1}H-NMR (400 MHz, D_2O): δ 1.62 (d, *J* = 7.3 Hz, 3H, H3), 2.82 (s, 3H, N-CH_3), 5.12 (q, *J* = 7.4 Hz, 1H, H2), 7.64 (t, *J* = 7.9 Hz, 2H, H3' & H5'), 7.80 (t, *J* = 7.5 Hz, 1H, H4'), 8.03 (d, *J* = 8.0 Hz, 2H, H2' & H6'); **^{13}C-NMR (101 MHz, D_2O)**: δ 15.2 (q, C3), 30.9 (q, N-CH3), 59.6 (d, C2), 128.9 (d, C2' & C6'), 129.3 (d, C3' & C5'), 132.3 (s, C1'), 135.4 (d, C4'), 197.7 (s, C1) ppm; m.p. 179-180 °C, (R): α_{D20} = +48.3° (c 0.7, H2O), 94% ee, (S): α_{D20} = -51.5° (c 0.8, H2O), 97% ee; HPLC method: Heptane/IPA = 96.9/3 + 0.1% DEA, 1mL/min, 25 °C, AS-H (t_S= 8.0 min, t_R= 11.1 min).

(R)/(S)-2-(Methylamino)-1-(4-(trifluoromethyl)phenyl)-propan-1-one hydrochloride

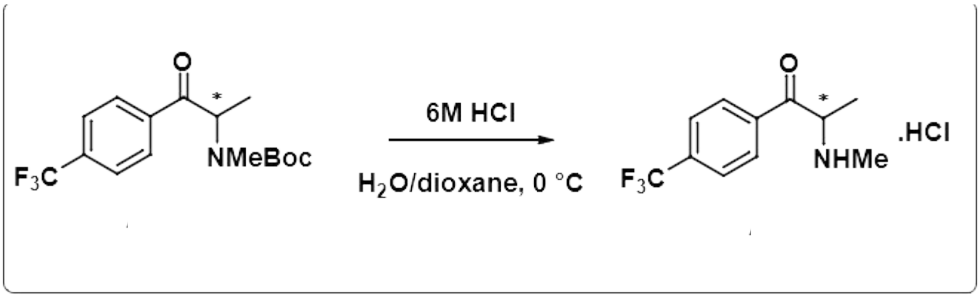

The compound was prepared according to **General procedure C**. The desired compound was obtained as colorless solid in **83%** (R), resp. **83%** (S) yield.

^{1}H-NMR (400 MHz, D_2O): δ 1.62 (d, *J* = 7.3 Hz, 3H, H3), 2.84 (s, 3H, N-CH_3), 5.16 (q, *J* = 7.3 Hz, 1H, H2), 7.94 (d, *J* = 8.2 Hz, 2H, H2' & H6'), 8.17 (d, *J* = 8.1 Hz, 2H, H3' & H5'); **^{13}C-NMR (101 MHz, D_2O)**: 14.8 (q, C3), 30.9 (q, N-CH_3), 59.9 (d, C2), 123.5 (q, $^{1}J_{C\text{-}F}$ = 272.2 Hz, CF_3), 126.2 (dq, $^{3}J_{C\text{-}F}$ = 3.7 Hz, C3' & C5'), 129.4 (d, C2' & C6'), 135.1 (q, $^{2}J_{C\text{-}F}$ = 32.7 Hz, C4'), 135.3 (s, C1'), 196.8 (s, C1) ppm; m.p. decomposition >200 °C, (R): αD20 = +38.0° (c 0.87, MeOH), >98% ee, (S): αD20 = -34.9° (c 0.87, MeOH), >97% ee; HPLC method: Heptane/IPA = 98.9/1 + 0.1% DEA, 1mL/min, 25 °C, AS-H (t_S= 9.6 min, t_R= 11.4 min).

Sample Sizes

Figure 1

	MC	MC	4-MC	4-MC	4-MMC	4-MMC	4-TFMMC	4-TFMMC
	S	R	S	R	S	R	S	R
DAT	4	4	5	5	4	5	5	4
SERT	3	3	4	4	3	3	3	3

Number of independent experiments performed in triplicate

Figure 2

Panels b-I, [^{3}H]5-HT release

	MC		4-MC		4-MMC		4-TFMMC	
Conc [µM]	S	R	S	R	S	R	S	R
1	6	3	10	6	8	6	9	3
3	6	3	10	7	11	9	11	5
10	17	8	10	9	11	14	12	16
30	13	4	10	7	17	22	16	19
60	3	6				12		9
100	11	8	10	5	8	17	9	16
300	10	8				11		
600		5				8		
1000	9	3						

Panels o - r, electrophysiology

	MC		4-MC		4-MMC		4-TFMMC	
Conc. [µM]	*S*	*R*	*S*	*R*	*S*	*R*	*S*	*R*
0.1					4			
0.3		3	5		3	4	5	7
1	5	3	5	3		5	5	7
3	5	3	5	4		4		7
10	5	3	4	4	4	5	5	7
30	5	4	4	4	4	5	5	7
100	5	4	5	4	4	5	5	7
300	5			4				

Figure 3

Panel		vehicle	4-MC			4-MMC			4-TFMMC		
	mg/kg	N/A	1	5	10	1	5	10	1	5	10
3a		12	7	7	8	7	8	7	7	8	8
3b		12	8	7	7	8	7	8	8	7	6
3c		13	7	7	9	7	7	7		7	7
3d		9	6	6	6	6	6	6		6	6
3e		13	7	7	9	7	7	7		7	7
3f female		10		10			10				10
3f male		10		10			10				10

Figure 4

Panel		vehicle	D-FEN (3mg/kg)	D-FEN (10 mg/kg)	S-4-MC	S-4-MC+FLX	S-4-TFMMC	S-4-TFMMC + FLX	R-4-MC	R-4-TFMMC	FLX
b and c		4	4	4							
h		5	5		5	5	5	5	5	5	5
j-l (5-HT)					5	5					
j-l (DA)					4	4					

Suppl Figure 1

	MC		4-MC		4-MMC		4-TFMMC	
µM	S	R	S	R	S	R	S	R
1	6	3	10	6	8	6	9	3
3	6	3	10	7	11	9	11	5
10	17	8	10	9	11	14	12	16
30	13	4	10	7	17	22	16	19
60	3	6				12		9
100	11	8	10	5	8	17	9	16
300	10	8				11		
600		5				8		
1000	9	3						

Suppl Fig 2

Panels		S-4-MC	S-4-TFMMC	MDMA	S-4-MMC
a		10	7	7	8
	1µM				
	5HT2A	9	9		
	5HT2B	6	6		
	5HT2C	8	8		
	10µM				
	5HT2A	9	9		
	5HT2B	6	6		
c	5HT2C	8	8		

Suppl Fig 3
N=15 per condition

Suppl Fig 4
N=4 per condition

Suppl Fig 5
N=5 per condition

Suppl Fig 6
N=3 per condition

Suppl Fig 7
N=3 per condition

Suppl Fig 8
N=3 per condition
