# Supplementary material for: Serotonin-releasing agents with reduced off-target effects
Source: Mol Psychiatry. 2022 Nov 9;28(2):722–32. doi: 10.1038/s41380-022-01843-w (PMC9645344; doi:10.1038/s41380-022-01843-w)
Supplement: Supplementary file 2 — Supplementary Information Figure Legends [file 41380_2022_1843_MOESM2_ESM.docx]

**Suppl. Figures**

**Suppl. Fig. 1: Effect of the stereoisomers of MC, 4-MMC and 4-TFMMC on SERT-mediated efflux.** Shown are the effects of the indicated *S*- **a through c)** and *R*-enantiomers **d through f)** of the individual cathinones. Data are shown as mean and standard deviation. **g)** correlation analysis for maximal induced [^3^H]5-HT efflux versus maximal induced currents of test drugs. The linear regression fit is displayed as a red line with 95% confidence intervals (*R*^2^ = 0.9661; *F* = 170.9; *P* < 0.0001). *N*>3 independent observations per compound and concentration. Detailed sample sizes are given in the supplementary information.

**Suppl. Fig. 2: Effect of *S*-4-MC, *S*-4TFMMC, *S*-4-MMC and MDMA on VMAT mediated uptake and 5-HT receptors. a)** Uptake of tritiated 5-HT into vesicles in PC12 cells via endogenously expressed VMATs was determined in presence of the indicated drugs (all at 30 µM) and their inhibitory potency was expressed in relation to the inhibition observed in presence of 30 µM reserpine. ★ denotes *P* ≤ 0.05 versus MDMA. Kruskal-Wallis (Dunn’s test). **b)** representative trace of substance evoked (*S*-4-TFMMC and *S*-4-MC at 100 nM, 1 µM and 10 µM; 5-HT at 1 µM) change in GCamP6s fluorescence in HEK293 cells expressing the 5HT2A receptor **c)** response to indicated drugs (1 µM or 10 µM respectively) in HEK 293 cells expressing the indicated 5HT2 receptors, measured by increase of fluorescence intensity of the Ca^2+^ sensor GCamP6s normalized to the response evoked by 1 µM 5-HT. All data are shown as mean and standard deviation. *N*= at least 6 independent observations per condition. Detailed sample sizes are given in the Supplementary Information.

**Suppl. Fig. 3: Effect of the stereosisomers of 4-MC, 4-TFMMC and 4-MMC on surface expression of DAT and SERT.** HEK293 cells stably expressing CFP-tagged human SERT or DAT were exposed to the indicated test-drugs for 1 hour (as described in the methods section). Data are shown as mean and standard deviation. Conditions were compared with Kruskal-Wallis test and multiple comparisons conducted with Dunn’s test. Scale bar: 10 µM. *N*=15 observations per condition.

**Suppl. Fig. 4: Effect of systemic D-FEN on extracellular 5-HT in individual animals**

Shown is the relative change in serotonin-sensor derived fluorescence following intraperitoneal administration of saline (SAL), or D-FEN at 3 or 10 mg kg^-1^ at t=120 s in 4 different animals.

**Suppl. Fig. 5: Effect of saline and drugs of interest on extracellular 5-HT in individual animals.** Shown are the effects of saline (SAL) and the indicated drugs on the relative change in 5-HT sensor-based fluorescence in 5 individual animals. Drugs were administered at t=600 s (intraperitoneal injection). The different colours reflect individual animals. FLX: 10 mg kg^-1^ / D-FEN: 10 mg kg^-1^/ *S-*4-MC: 5 mg kg^-1^/ *S-*4-TFMMC: 10 mg kg^-1^ / *R-*4-MC: 5 mg kg^-1^/ *R-*4-TFMMC: 10 mg kg^-1^.**Suppl. Fig. 6 *S*-4-MC induced elevation in extracellular 5-HT on two consecutive days.**

**a)** Representative traces displaying changes in 5-HT-sensitive fluorescence in the NAc following two separate administrations of *S*-4-MC (5 mg kg^-1^, intraperitoneal injection at t= 0 s) 24 hours apart in 3 individual animals. **b)** relative 5-HT-sensitive fluorescence at t= 500-600 s post-injection, normalized to day 1 (data for day 2 are shown as mean and standard deviation). N=3. Data were analyzed using Wilcoxon matched-pairs signed rank test.

**Suppl. Figure 7: Effect of systemic *S*-4-MC and *S*-4-TFMMC on extracellular dopamine in the NAc**

**a)** Shown is the relative change in fluorescence of a genetically encoded sensor for dopamine following the intraperitoneal injection of saline, *S*-4-MC (5 mg kg^-1^), *S*-4-TFMMC (10 mg kg^-1^) or the dopamine releasing agent d-amphetamine (AMPH; 5 mg kg^-1^) in 3 different animals. The arrow indicates the time point of the injection (=120 s).

**b)** Shown is the relative fluorescence at t=800-1000 s for each drug treatment depicted in a). *N*=3, ★ denotes *P* ≤ 0.05 versus saline; Kruskal-Wallis, Dunn’s multiple comparison test.

**Suppl. Figure 8: *S*-4-TFMMC-induced SERT-mediated 5-HT efflux *in vivo***

**a)** Cartoon depicting guide cannula (blue column) and active microdialysis membrane (red tip, active length of 1mm) placement in the NAc. **b)** effect of *S*-4-TFMMC (10 mg kg^-1^, intraperitoneal injection) on extracellular 5-HT in presence or absence of co-administered fluoxetine (FLX, 10 mg kg^-1^). **c)** total area under the curve (A.U.C.) of the traces shown in **b**. *N*=3,★ denotes *P* ≤ 0.05 (one-tailed Mann-Whitney test). Data are shown as mean and standard deviation.
