# Supplementary figures and images for: Serotonin-releasing agents with reduced off-target effects
Source: Mol Psychiatry. 2022 Nov 9;28(2):722–32. doi: 10.1038/s41380-022-01843-w (PMC9645344; doi:10.1038/s41380-022-01843-w)

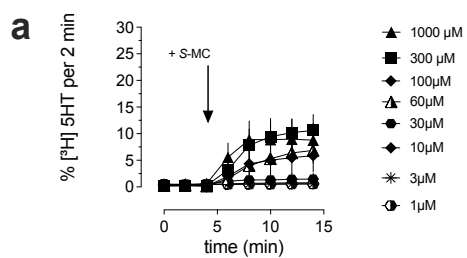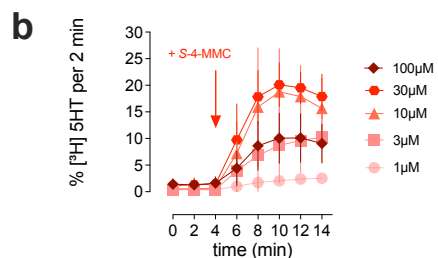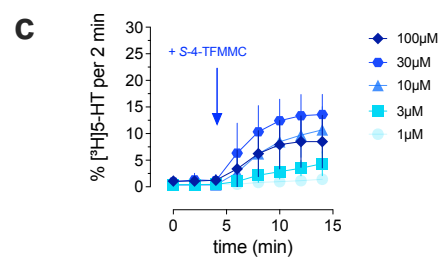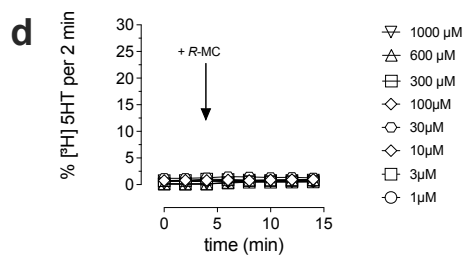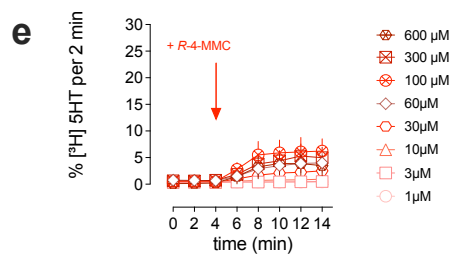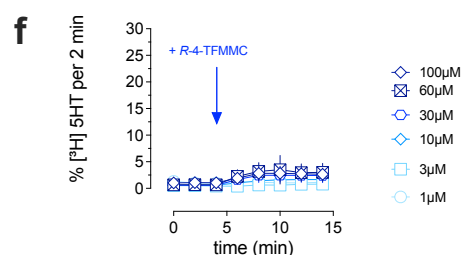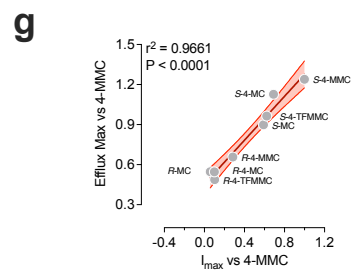

Supplement: Supplementary file 3 — Supplementary Figure 1 [file 41380_2022_1843_MOESM3_ESM.pdf]

**a**

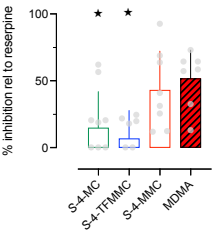

**b**

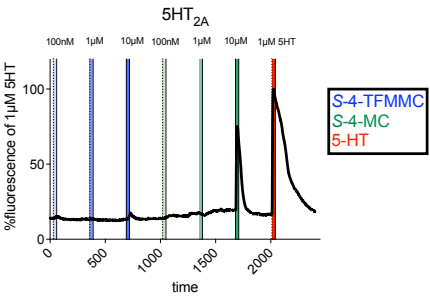

**c**

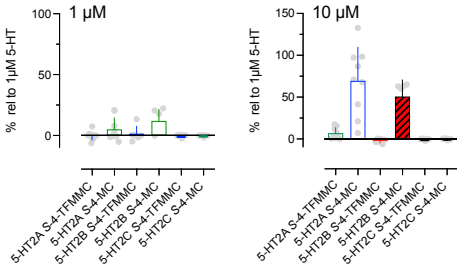

Supplement: Supplementary file 4 — Supplementary Figure 2 [file 41380_2022_1843_MOESM4_ESM.pdf]

SERT

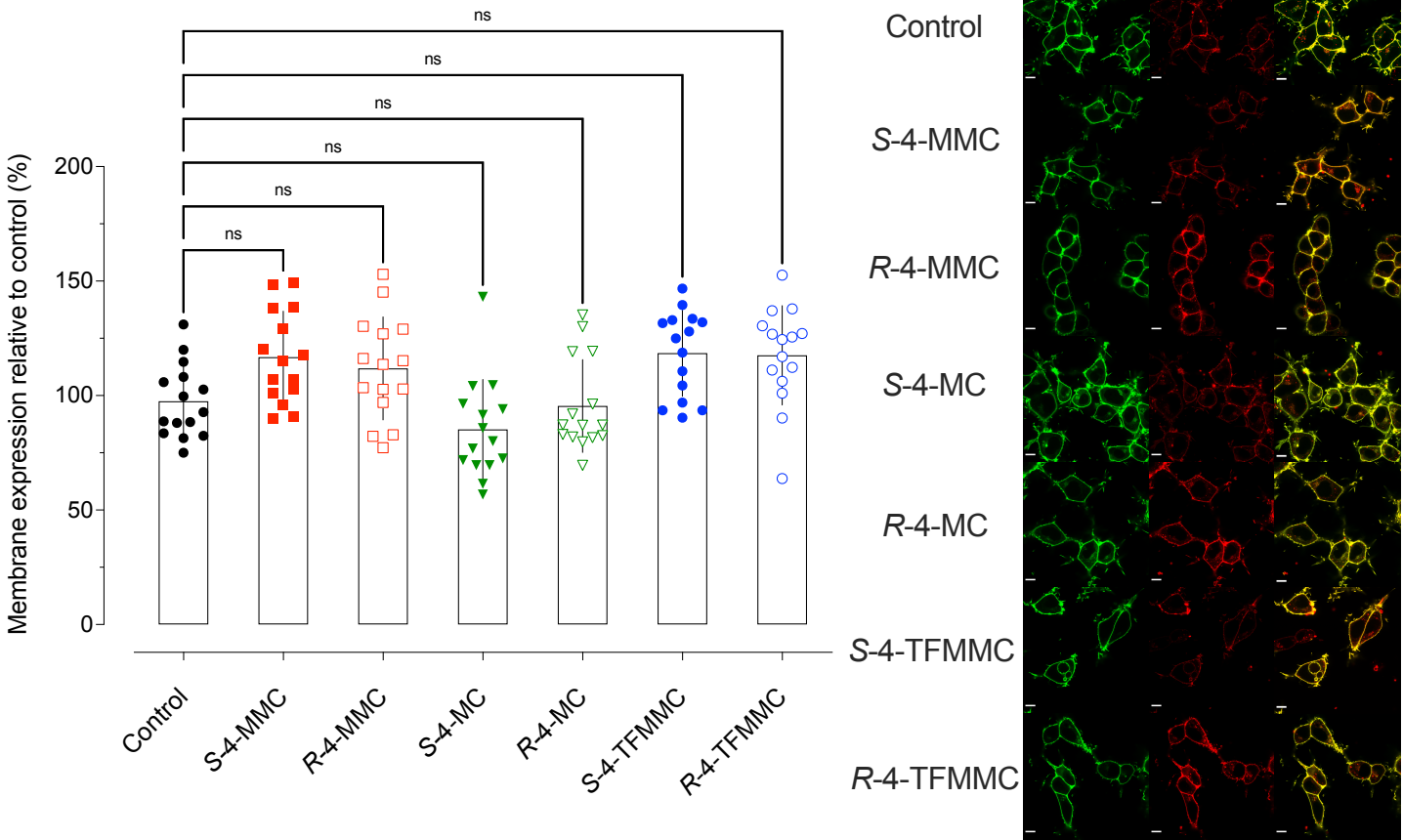

DAT

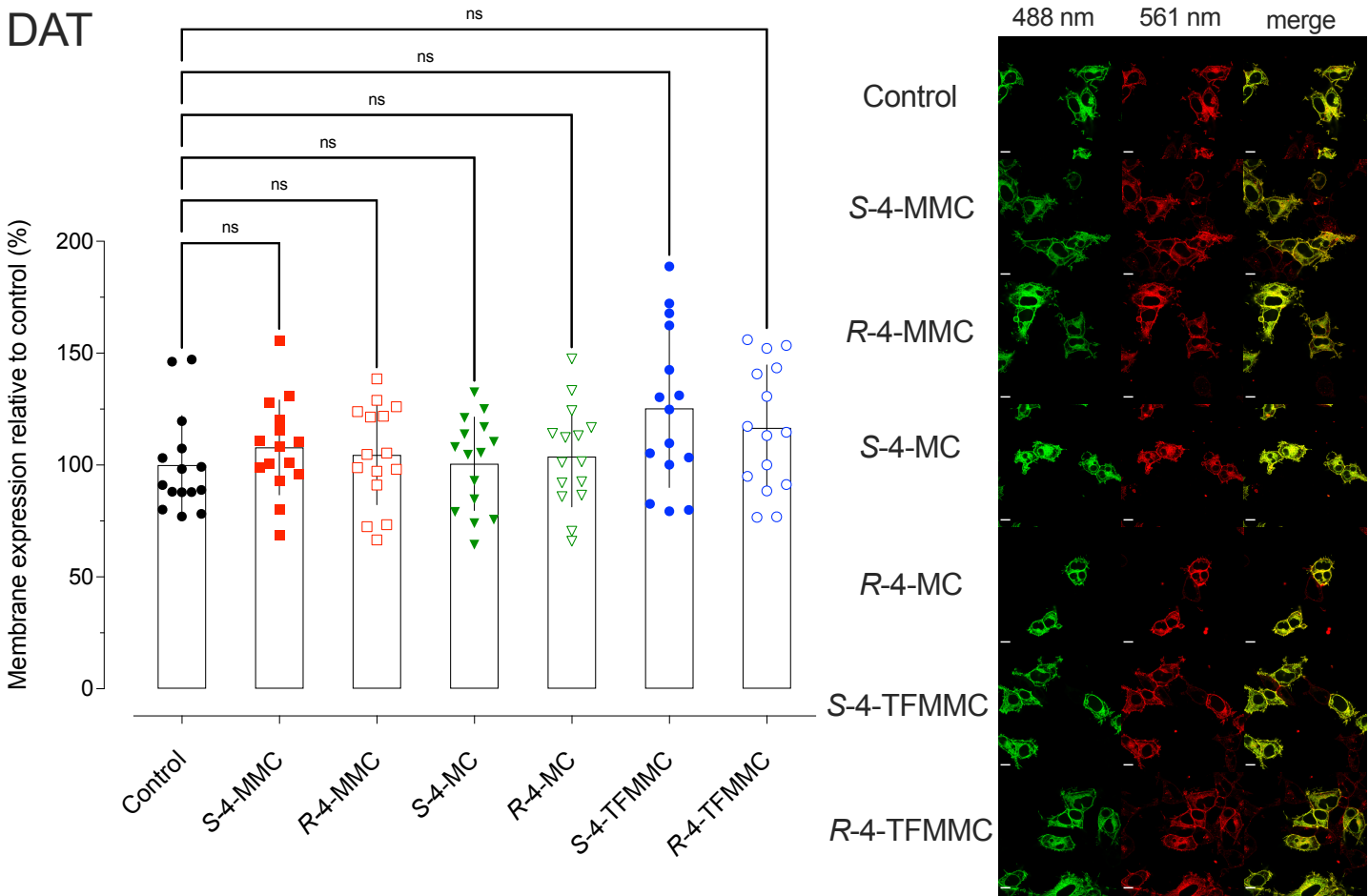

Supplement: Supplementary file 5 — Supplementary Figure 3 [file 41380_2022_1843_MOESM5_ESM.pdf]

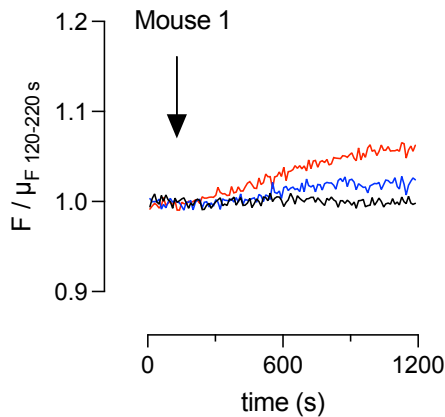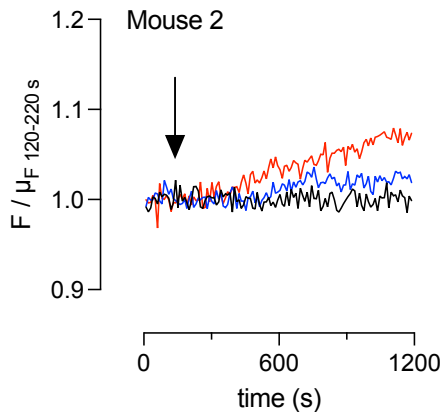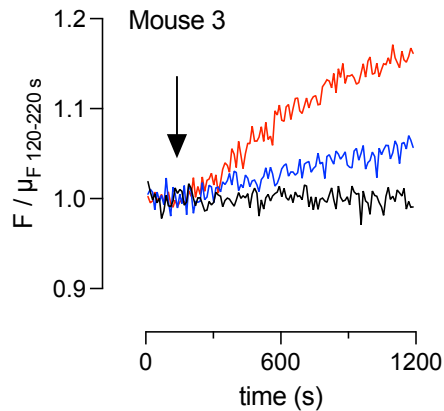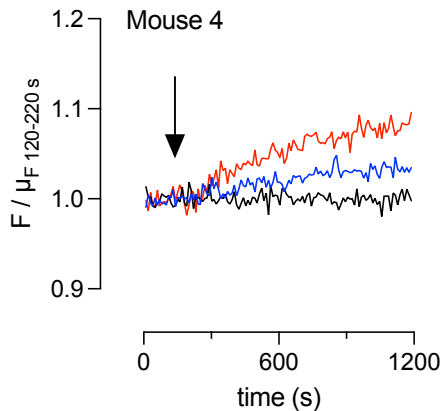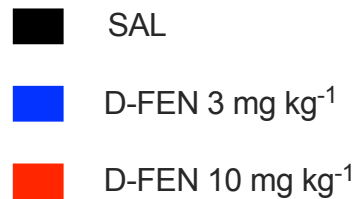

Supplement: Supplementary file 6 — Supplementary Figure 4 [file 41380_2022_1843_MOESM6_ESM.pdf]

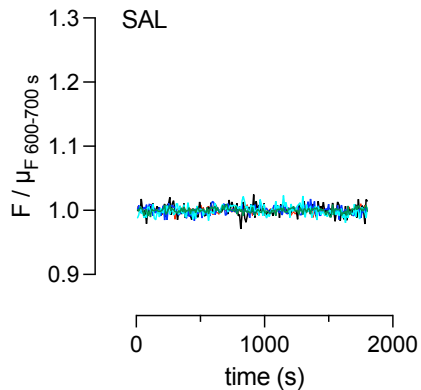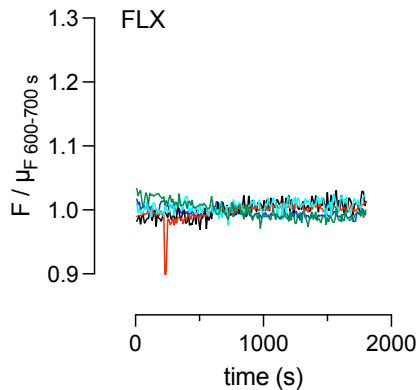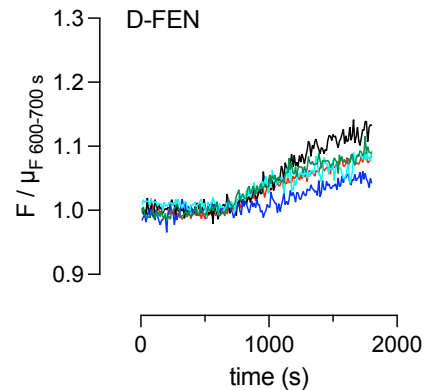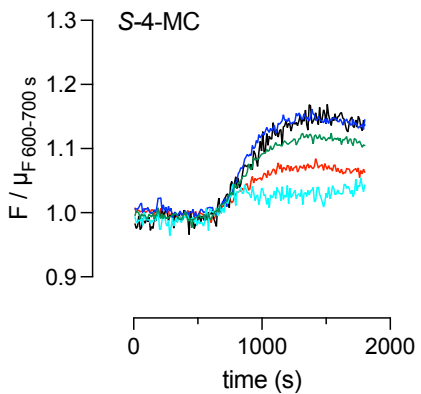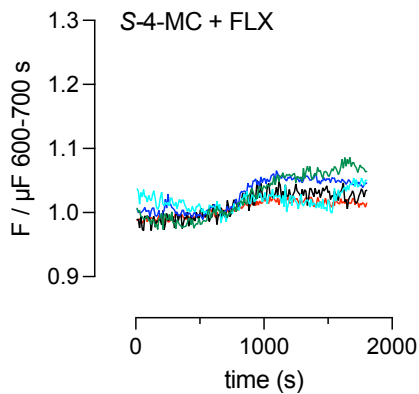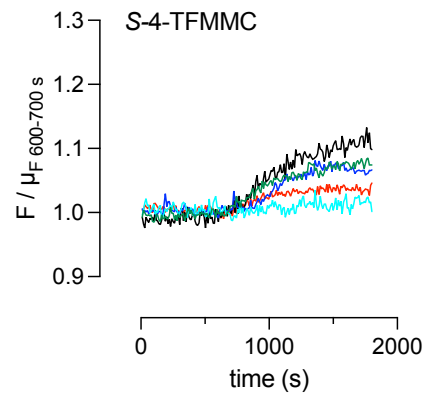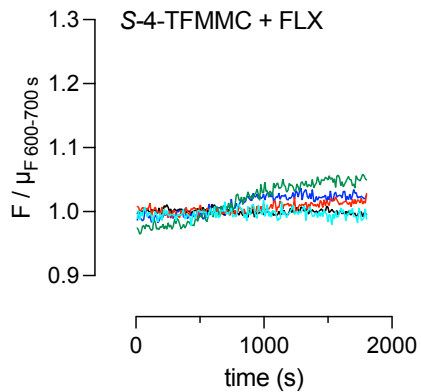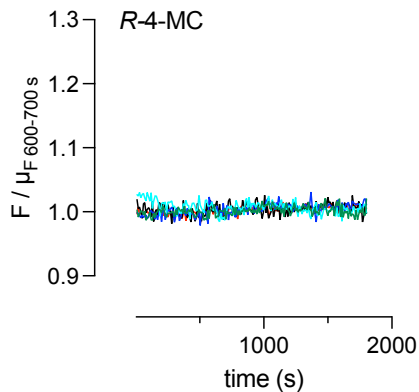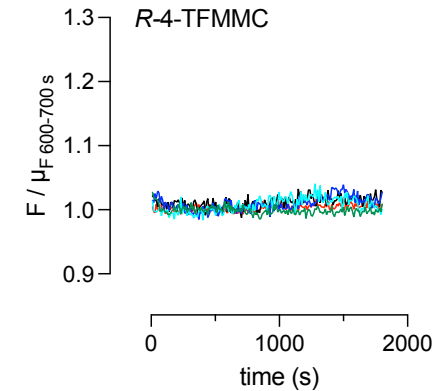

Supplement: Supplementary file 7 — Supplementary Figure 5 [file 41380_2022_1843_MOESM7_ESM.pdf]

**a**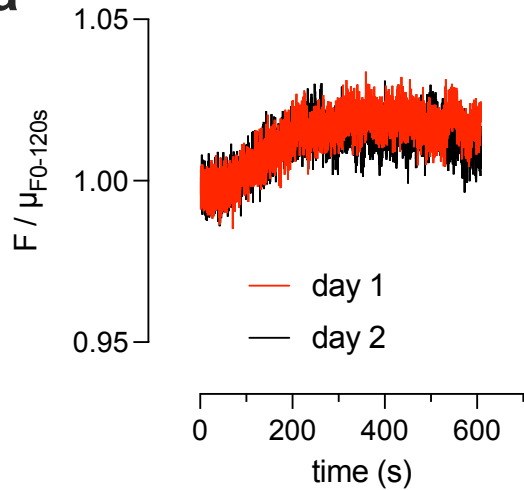**b**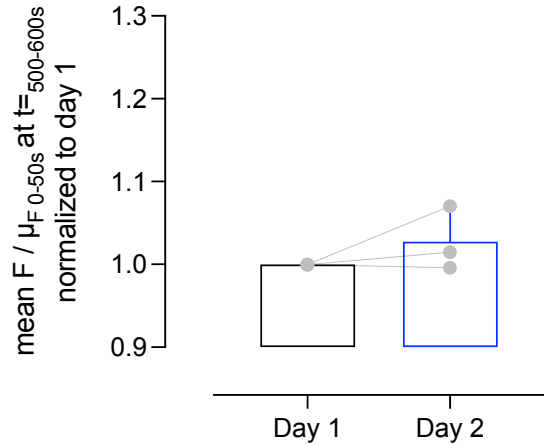

Supplement: Supplementary file 8 — Supplementary Figure 6 [file 41380_2022_1843_MOESM8_ESM.pdf]

**a**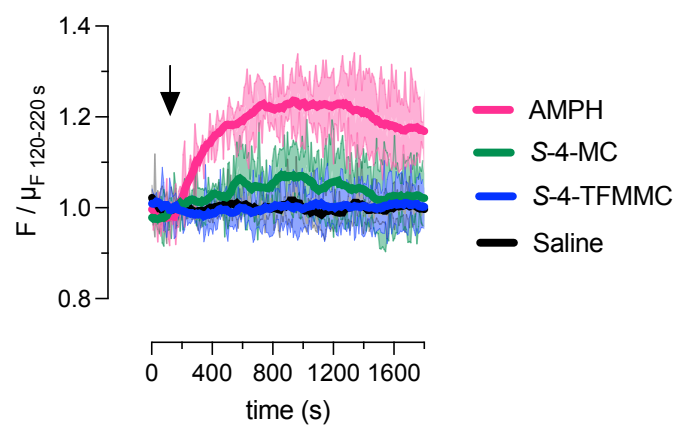**b**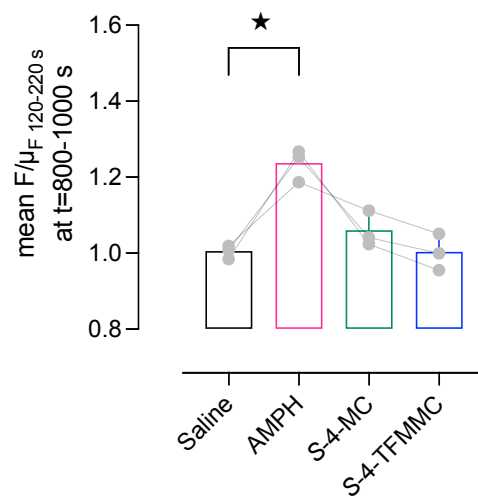

Supplement: Supplementary file 9 — Supplementary Figure 7 [file 41380_2022_1843_MOESM9_ESM.pdf]

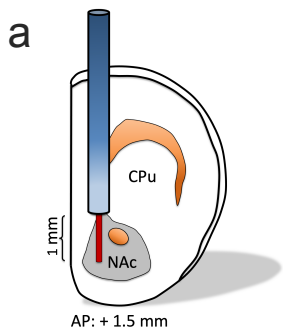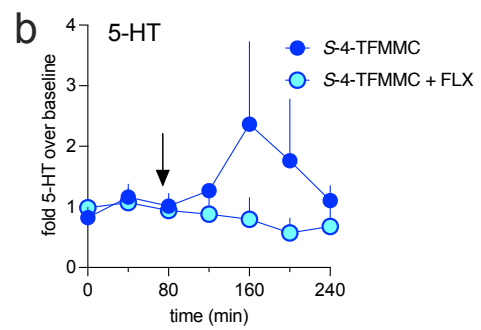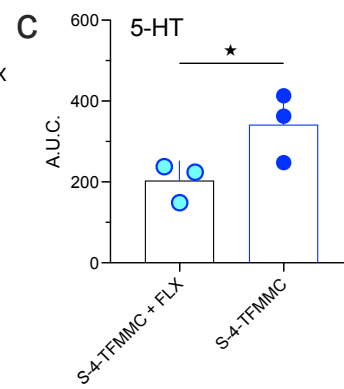

Supplement: Supplementary file 10 — Supplementary Figure 8 [file 41380_2022_1843_MOESM10_ESM.pdf]
